# Recent Advances in Membrane-Based Air Filtration Technologies for Ambient Particulate Matter Separation

**DOI:** 10.3390/polym17243265

**Published:** 2025-12-09

**Authors:** Prarthana Bora, Chinmoy Bhuyan, Duraikkanu Shanthana Lakshmi, Swapnali Hazarika, Marek Tanczyk, Srinivas T. G. Srimath

**Affiliations:** 1 Chemical Engineering Group and Centre for Petroleum Research, CSIR-North East Institute of Science and Technology, Jorhat 785006, India; 2 Academy of Scientific and Innovative Research (AcSIR), Ghaziabad 201002, India; 3 RSK Environment Ltd., 18, Frogmore Road, Hemel Hempstead HP3 9RT, UK; sri.gudimella@tetratech.com; 4 Institute of Chemical Engineering, Polish Academy of Sciences, 44-100 Gliwice, Poland; mtanczyk@iich.gliwice.pl; 5 Tetra Tech Limited, Sovereign Street, Leeds LS1 4ER, UK

**Keywords:** particulate matter, air pollution, membrane, air filter

## Abstract

Varied types of particulate matter (PM) persist in the environment and exert a harmful impact on public health. The aim of this review article is to explore the key role of membrane technology in the separation of PM from ambient air. Nanofibrous, microporous, Janus, photocatalytic and hollow fiber membranes have found significant utilization in the effective separation of PM. Recent advancements in membrane technology and their key properties such as antibacterial activity, flame retardancy, wettability, thermal stability and reusability have been underscored in this review article. Moreover, the principles of PM separation have been discussed in detail to understand the working pathway of a membrane air filter via physical, chemical or biological approaches. A brief comparison between the conventional air filters and membrane air filters is provided in terms of cost, separation principle and respective merits and demerits to understand the importance of membranes in the realm of PM separation. This study also highlighted the commercial status of PM air filters with respect to their cost and scalability. By focusing on the innovations in membrane filters, this review article has highlighted the futuristic approaches such as green fabrication techniques, highly efficient material incorporation, use of AI/ML, etc., to overcome the challenges associated with conventional air filters.

## 1. Introduction

Particulate matters (PMs) represent a class of persistent air pollutants which possess damaging impacts on the environment as well as human health. Air pollutants are divided mainly into two categories, namely primary and secondary. Ozone, greenhouse gases, VOCs and airborne PM are classified as secondary air pollutants. They tend to exert high health risks and can cause serious chronic as well as acute effects on human cardiovascular and respiratory systems [1]. PM in fine form is even more dangerous to human health [2]. PM is observed as a yellowish and murky haze that quilts the sky from sunlight. This type of event significantly affects and alters the regional climate. PM also taints water bodies such as rivers and lakes, disrupting their ecosystems and wreaking havoc on forests, agriculture, and ecological systems [3,4]. Alzheimer’s, neurological diseases, pulmonary disease, ischemic heart disease, acute lower respiratory infections, stroke, and lung cancer are found to have occurred frequently upon PM exposure, leading to increased mortality rates. Henceforth, there arises an urgent necessity to devise timely interventions aimed at ameliorating or reducing PM exposure within the environment. The traditional approaches for air pollution remediation include mechanical filtration, high-efficiency particulate air (HEPA) filtration, activated carbon (AC) technology, ultraviolet (UV) technology, negative ionization, and ozone treatment [5].

**Figure 1:**
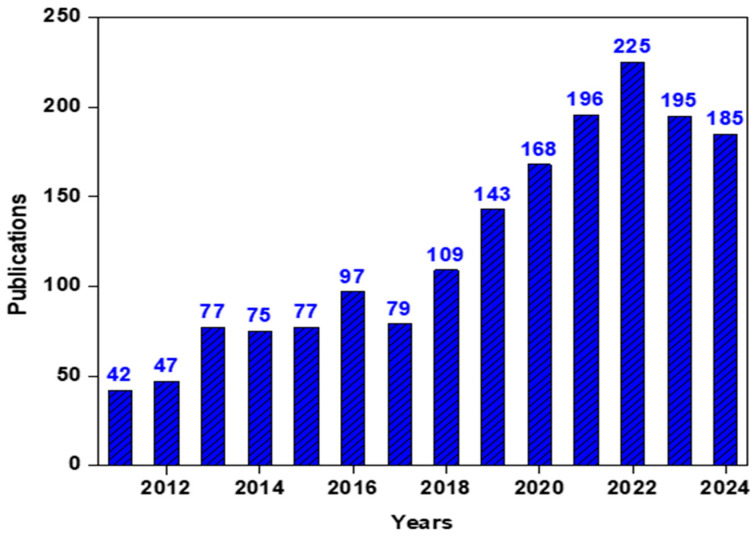
Publication record in the period of 2011–2024 from Web of Science database with keywords ‘Membrane’ and ‘Particulate matter’ (accessed on 12 February 2025).

Membranes can be defined as semipermeable barriers with the advantages of controllable pore size, flexibility, tunable surface functionality, durability, high selectivity, small footprint and easy fabrication [6,7,8]. Recently, numerous efforts have been made in the development of nanofibrous membranes modified with various functional materials specifically for air filtration [9,10,11,12]. The performance of a membrane primarily depends on its pore structure, size, and surface functionalities. A wide variety of air filter membranes are found in the literature, such as nanofibrous membranes, microporous membranes, Janus membranes, photocatalytic membranes, hollow fiber membranes, etc., which can be differentiated on the basis of their pore structure, properties, inner texture and fabrication processes. Membranes can be engineered with tailored functionalities to demonstrate high filtration efficiency, remarkable thermal and mechanical resilience, potent antimicrobial activity, favorable wettability and photocatalytic activity. Owing to these advantages, the demand for membrane technology in air filtration applications has been growing day by day. A trend in publications during the period 2011–2024, as sourced from the Web of Science database, using the keywords ‘Membrane’ and ‘Particulate matter’, is illustrated in Figure 1. The data clearly indicate a significant increase in the popularity of membranes for the removal of PM components over the past decade.

Many reviews have been reported in recent years emphasizing advancement in PM filtration technologies. Ji et al. have reviewed the fundamentals of PM, sources, types, mechanisms, etc., along with existing materials and their properties for PM separation [13]. Zhou et al. have reviewed the advances in electrospun nanofiber membranes in the field of air filtration. This study has highlighted the principle of electrospinning, filtration principles and performances of electrospun membranes [14]. Reccardis et al. [15] and Valencia-Osorio et al. [16] have reported reviews that discuss the recent literatures on electrospun nanofiber membranes for air filtration. Lu et al. have discussed the application of electrospun nanofibers in air filtration and the impacts of fiber structure on filtration efficiency and pressure drop [17]. Natural polymer-based electrospun membranes for air filtration have also been reviewed with their characteristics, performances and modification strategies [18]. Most of the reviews reported are limited to only electrospun membranes and their properties, principles and performance. Some of them have only focused on materials of air filters and their characteristics. Despite the availability of several reviews, a clear literature gap can be observed. A review article covering PM classifications, sources, existing air filters, separation principles, air filter materials, diverse membranes and their properties is still lacking. This review addresses all these aspects while discussing recent advancements in membrane technology (beyond electrospun nanofiber membranes) for separation of PM components from ambient air. Conventional air filters and membrane-based air filters relevant to PM separation are discussed with their associated merits and demerits. Special features of membranes like antimicrobial activity, wettability, thermal stability, flame retardancy and reusability are highlighted, underscoring their impact in the generation of high-quality air filters. Moreover, no review paper has discussed air filters from a commercial point of view to date. This review discusses the existing commercial air filters, their availability and challenges. A comparative discussion among various membranes and materials along with their advantages and disadvantages is another gap that the current review has covered. This comparative discussion can be used as a tool for strategic modification of properties as well as the performance of existing air filters for future research. In the future prospect section, this review article addresses the potential materials, green fabrication methods, and inclusion of advanced technologies such as AI/ML for membrane design.

## 2. PM and Its Types

PM represents a complicated aerosol mixture present as a major component in polluted air [19]. The most common components of PM are organic and elemental carbon along with inorganic matter (SO_4_^2−^, NO_3_^−^, Cl^−^, SiO_2_, heavy metals, etc.). PM is found as tiny droplets of liquid with suspended solid particles [20]. The chemical components present in PM may vary, generating three general categories: oily/non-oily particles, bioaerosols and non-biological aerosols [21]. Dioctyl phthalate and NaCl are generally considered as oily and non-oily components of a model PM pollutant, respectively [15]. Again, PM can be reclassified as solid particles and wetting and non-wetting liquid droplets based on physical state and wettability [22]. Aerodynamic diameter is another important property depending on which PM components can be categorized as coarse particles with size > 2.5 μm (for example, PM_10_), fine particles with size ≤ 2.5 μm (for example, PM_2.5_ and PM_0.3_) and ultrafine particles of size ≤ 0.1 μm (for example, PM_0.1_) [23]. Fine PM (PM_2.5_, PM_0.3_ and PM_0.1_) can penetrate through the alveoli and affect the cardiovascular (CV) function. When fine PM contaminated with bacteria and viruses enters the respiratory system, the health risk significantly increases [24,25]. Due to growing concern about the PM hazards, many countries have established PM monitoring systems especially for PM_2.5_ and PM_10_. Simultaneously, various products for protection against PM components of different aerodynamic properties are available [26,27]. This study summarizes the membrane-based separation strategies for PM components of all sizes, including PM_0.1_, PM_0.3_, PM_2.5_, and PM_10_.

**Figure 2:**
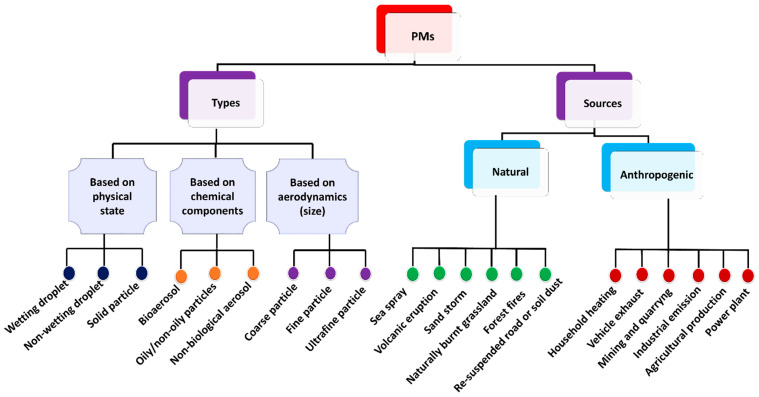
Types and common sources of PM.

PM originates from both natural phenomena and human activities. The types as well as the common sources of PM are depicted in Figure 2. Natural sources of PM encompass sandstorms, sea spray, volcanic eruptions, soil dust, forest fires, and naturally burnt grasslands [19]. However, anthropogenic activities such as industrial emissions, agricultural practices, household heating, incomplete combustion of fossil fuels, construction dust, vehicle emissions, and mining and quarrying operations contribute predominantly to PM pollution [28,29]. Based on the formation mechanism and sources of PM, two separation methodologies are proposed, i.e., source control and end-of-pipe process [30].

## 3. Influence of PM on Health and the Environment

**Figure 3:**
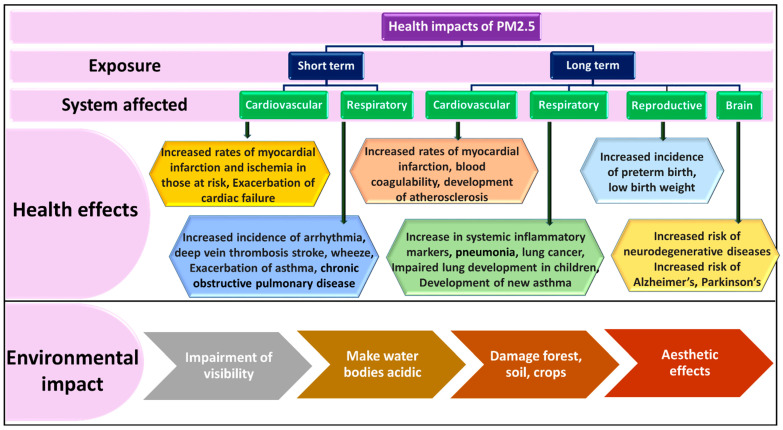
Health and environmental impacts of PM_2.5_ exposure [44].

The adverse effects of fine PM with a diameter of ≤2.5 μm on human health are firmly established [31,32]. Both the European Society of Cardiology and the American Heart Association have identified PM_2.5_ as a significant hazard to CV health, emphasizing the immediate requirement for effective strategies to mitigate PM exposure [33]. Evidently, HEPA filtration is able to decrease PM_2.5_ from indoor air, making a significant contribution towards CV health [34]. Even at very minimal concentration, PM_2.5_ can be fatal. Reportedly, fine particle exposure, which may be from ambient fine particles or originated from tobacco or cooking smoke, may cause premature death [35]. People in developing countries and rapidly growing urban areas are mostly affected by PM exposure [36]. The Reducing Air Pollution in Detroit Intervention Study (RAPIDS) aimed to assess the efficacy of air filtration in diminishing personal-level PM_2.5_ exposures and alleviating associated CV health impacts among the elderly in a typical urban setting in the United States [37]. In 2017, the Global Burden of Disease Study revealed that ambient particle pollution, including nanoparticle pollution, ranked among the top four risk factors for mortality and disability-adjusted life years (DALYs) nationally in China, experiencing the second-highest increase (88.5%) from 1990 to 2017 [38]. Recently, the COVID-19 pandemic has resulted in widespread severe respiratory illness globally [39]. Emerging evidence recommends that the transmission of coronavirus (SARS-CoV-2) occurs in public spaces through PM aerosol particles [40,41]. PM pollution contributes to widespread haze which can obscure sunlight and diminish its penetration through the atmosphere. These fine particles, when combined with infrared-absorbing greenhouse gases, contribute to approximately half of the greenhouse effect and reflect a significant portion of incoming sunlight back into space. As a result, PM pollution not only poses severe risks to public health but also has profound environmental effects, including reduced visibility, disruption of ecosystems, climate alterations, and changes in radiative forcing [42]. On hazy days, the impact of PM is starkly evident, with the cityscape obscured by a dense veil of pollution [42]. Moreover, with increases in the PM level in the environment, decreases in biodiversity as well as ecosystem goods and services are witnessed [43]. Other consequences of PM level enhancement are acid rain, damage to crops and forests, depletion of nutrients in soil, imbalances of nutrients in coastal water, acidic lakes and streams, etc. Figure 3 shows various health and environmental effects caused by PM_2.5_ exposure. In light of these impacts, the WHO has determined exposure limits for PM. Additionally, the permissible exposure limits on a daily and annual basis under NAAQS (India) and those adopted in the UK are given in Table 1.

## 4. Conventional Methods of PM Separation Versus Membrane

Mechanical air filters are widely used for air filtration and they separate the pollutants based on their pore sizes [45]. A HEPA filter is a type of mechanical air filter consisting of pleated paper integrated with densely packed glass fibers. HEPA filters work via various mechanisms, viz.: interception, impaction and diffusion. They have achieved PM_0.3_ removal efficiency of 99.97% with an air flow rate of 150 to 400 cubic feet per minute [46]. Lifetime is a very important property to consider when designing an air filter. HEPA filters often involve the incorporation of pre-filters to increase the lifetime. Pre-filters are generally fiber meshes having large pores which trap bigger particulates before reaching the HEPA filter while minimizing the possibility of pore blockage. Another traditional air filtration technique is the use of electronic filters. In this type of filters, an electric field is used to ionize the passing particulates. An electrostatic smoke precipitator (ESP) is one such example of an electronic filter which operates at high voltages [46]. ESPs can separate PM irrespective of its size and are found to outperform HEPA filters. Activated carbon (AC) is reported to offer high pollutant adsorption capacity due to its extensive surface area and porosity [47]. UV light purification is another method for addressing air filtration, but this method only deals with bacteria and pathogens [46]. Transparent filtration technology is a modern method of air filtration which utilizes various polymer materials [42]. Additionally, photocatalytic materials drive air purification via advanced oxidative reactions, degrading pollutants under specific wavelengths of radiation.

Membrane technology is highly advantageous due to its tuning ability to attain exceptional separation efficiency, antifouling ability, antimicrobial properties, etc. Mostly, nanofibrous (NF) membranes have been effectively used in the separation of airborne particulates ranging from the micron to sub-micron level. NF membranes possess small pore size, significant aspect ratio and superior pore density, which help in attaining high air filtration performance compared to other filtration processes. Table 2 represents the various methods available for air filtration and their properties like separation principles, cost, merits and demerits.

## 5. Strategic Approaches to Membrane Air Filter-Based PM Removal

### 5.1. Physical Approach

The physical approach is applicable in those PM separation membranes where no specific functionalities are there to interact with PM particles. Typically, physical separation of PM components occurs based on the sieving properties of the NF membranes, where passage of the PM components through the membrane is restricted. The filtration performance is influenced by factors such as the type of polymer used, the membrane’s basic weight, packing density, and the diameter and distribution of fibers within the membrane [49]. The main mechanisms that operate during physical filtration of PM components via membrane air filters are discussed below in two broad categories: passive trap and proactive capture.

#### 5.1.1. Passive Trap

**Figure 4:**
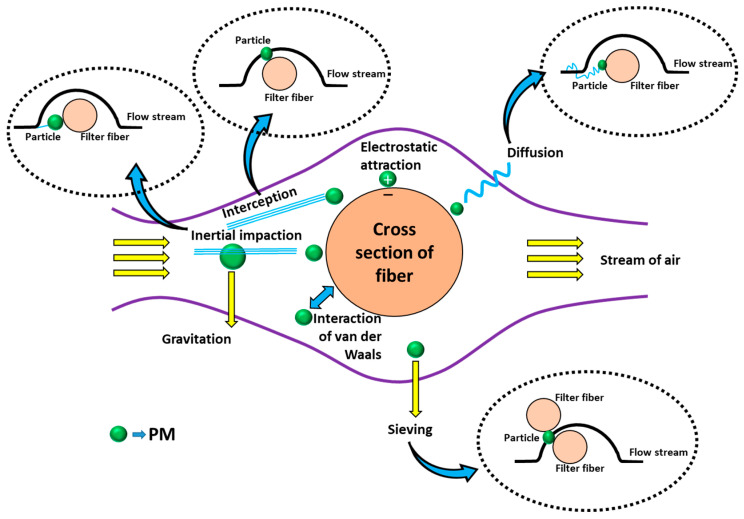
Different mechanisms during PM removal by physical approach.

Classical single-fiber filtration theories describe the PM passive trapping mechanisms. These passive trapping mechanisms involve interception, inertial impaction, diffusion, and gravity (shown in Figure 4). Interception allows PM to attach to the fiber physically. The interception mechanism is followed by particles of size up to 0.6 μm. PM components of size below 0.1 μm undergo diffusion-based separation [50]. Diffusion takes place when a particle touches the fiber under the act of a random motion. Reportedly, functional particles present in the nanometer range follow the diffusion mechanism and are captured [29]. The impaction mechanism utilizes the inertia concept and change in air flow path to isolate the PM components. In inertial impaction, larger particles (>0.3 μm) possessing sufficient inertia collide with filter fibers after breaking away from liquid streamlines [43]. Conversely, diffusion, driven by Brownian motion, guides smaller particles (<0.1 μm) away from air streamlines towards fiber adherence [51,52]. Additionally, PM can be passively trapped by gravity when airflow is perpendicular to the ground. The slip effect, which falls under the principle of diffusion and interception, is widely used to understand the flow of air molecules through a membrane. The slip effect generally arises from the slip of air through the fibers of an NF membrane, leading to a pressure drop. For example, an ultrathin 70 nm PVDF-based NF membrane has been developed which shows an excellent high PM_0.3_ filtering efficiency of 97.40% with a low pressure drop of 51 Pa, while the air flow is 5.3 cm/s. This superior result can be best explained from the synergistic interplay between the slip effect and ferroelectric dipole interaction [53]. The sieving mechanism operates when the spacing in between the fibers is smaller than the PM size. The fiber filter with smaller spacing prevents the passing of PM components and hence produces clean air. However, sieving filters often face durability issues. Over long periods of operation, the inner spaces of the overall filter become saturated with the in-depth loading of PM components, and clogging occurs [29].

If we consider a spider web as a real-world example, dust, pollen, insects, etc., sometimes collide with the spider web when they flow through the air and get trapped. This event is similar to the trapping of PM components in fibrous filters via various mechanisms like interception, inertial impaction, diffusion, and gravitation.

#### 5.1.2. Proactive Capture

Particulates of sizes varying from 0.05 to 0.5 mm cannot undergo diffusion or inertial impaction, which makes their separation more difficult [50]. In such cases, chemical or electrical forces are used to separate them via the proactive capture route [51]. Electrostatic separation requires ionizing wires for particle loading. The charged PM components are then diverted from the airflow streamline and become attached to the oppositely charged fiber surface through the electrostatic force of attraction, thereby resulting in the release of purified air to the ambient environment (Figure 4) [29,50]. This idea is applied in surgical masks and N95 respirators, which use charged melt-blown nonwovens as filter materials to collect PM, including bioaerosols. This idea makes use of the electrical forces, especially when the filter material is subjected to an external electric field or when the PM is charged [51]. One benefit of this proactive capture approach is that, in contrast to other approaches, it improves filtration efficiency without causing a pressure reduction [54]. For instance, Xie et al. used an ionizer to charge PM negatively and then gave carbonized cellulose aerogel a positive voltage [55]. Without causing a pressure decrease, this electric field force allowed the effective adsorption (>99.91%) of PM_2.5_ onto the aerogel. Conversely, conventional filtration materials that attempt to enhance filtration efficiency by means of increasing the thickness or packing density and lowering fiber diameter frequently cause appreciable pressure increases [13]. Applying an electric field to PM components or filter materials can multiply the electrostatic force of attraction, which consequently boosts the PM removal efficiency.

To understand proactive capture in a real scenario, we can consider the fibrous filter as a magnet. A magnet attracts magnetic subjects via exerting a magnetic field. Similarly, a fibrous filter of opposite charge can exert an electrostatic force of attraction and capture the targets.

### 5.2. Chemical Approach

Chemisorption is another approach via which effective separation of PM components can be achieved. In membranes involving chemisorption, PM components are adsorbed as a result of chemical interaction between PM components and membrane surface functionalities. Surface-modified air filters mostly involve this kind of separation approach. For example, a PVDF-based electret filter developed via grafting of SiO_2_ nanoparticles exhibits remarkable PM filtration performance [56]. Another such membrane is reported, where the polyetherimide (PEI) NF surface is modified with magnesium tetraphenylporphyrin (MgTPP) and a chelating agent ethylenediamine (EDA). This membrane shows very good filtration efficiency for PM_2.5_ and CO_2_ [57].

### 5.3. Biological Approach

The biological approach is very crucial as the risk of exposure to bioaerosols could results in various diseases. Although NF membranes can capture these bioaerosols effectively, they often produce secondary pollutants due to cumulative agglomeration. Thus, air filters having antimicrobial properties have gained enormous popularity. Antimicrobial properties can be introduced into a membrane by the incorporation of antimicrobial agents, which can be organic, inorganic or organic acids [58]. It is evident that some polymers like chitosan possess inherent anti-bacterial properties which can be used directly in the fabrication of anti-bacterial air filters [59].

**Figure 5:**
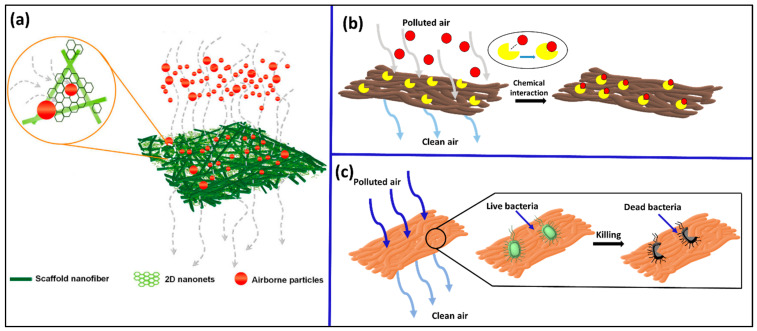
Air filtration via (**a**) physical sieving method. Reproduced with permission from ref. [60]. Copyright © 2017, Springer Nature. (**b**) Chemical approach and (**c**) biological approach.

A schematic representation of physical, chemical and biological approach of membrane air filters in removal of PM from polluted air is given in Figure 5.

## 6. Factors Considering the Performance of PM Filtration

### 6.1. Filtration Efficiency

Filtration efficiency provides the selectivity of a particular material for particulate removal, which can be calculated by using Equation (1).

(1) $$E_{PM\;}=\;\frac{C_{0}-C_{1}}{C_{0}}\times \;100\%$$

Here, *C*_0_ and *C*_1_ represent percentages of PM components on the filter before and after filtration, respectively. The concentration of PM components may be a mass concentration (μg m^−3^) or number concentration (m^−3^). *E_PM_* is the filtration efficiency, referred to as the key performance evaluation parameter of the PM separation filters. *E_PM_* is dependent on membrane (or filter) thickness, structure, airflow rate and porosity [13].

### 6.2. Pressure Drop

When assessing the air permeability or flow resistance of filter materials, pressure drop is frequently used [61]. The greater the pressure drop, the higher the fan’s power consumption will be. In small filtration devices like face masks, pressure drop directly influences breathability and personal comfort [62]. The pressure drop, i.e., *∆P* of an air filter membrane, can be measured from Equation (2).

(2) $$∆P=P_{0}-P_{1}$$

*P*_0_ and *P*_1_ are the feed pressure and permeate pressure in PM filtration, respectively.

### 6.3. Quality Factor

The quality factor (*QF*) provides an overall idea of an air filter/membrane system’s performance. The greater the QF value, the higher the performance. For QF measurements, Equation (3) can be utilized.

(3) $$QF=\frac{-ln\;ln\;(1-E_{PM})\;}{∆P}$$

*E_PM_* and *∆P* represent the filtration efficiency and pressure drop associated with the air filter or membrane, respectively [61]. From Equation (3), it is evident that the *QF* of an air filter is directly proportional to *E_PM_* and inversely related to *∆P*. Airflow rate significantly impacts filter performance, as higher velocities typically lead to a decrease in *E_PM_* and an increase in *∆P*. Consequently, the relationship between airflow velocity and filter performance is anticipated to be intricate. Han et al. coined a modified equation for the *QF* measurement by considering airflow velocity (Equation (4)) where *mQF* is modified *QF* and *V* is airflow velocity [63].

(4) $$mQF=\frac{-ln\;ln\;(1-E_{PM})\;}{∆P}V$$

### 6.4. Optical Transparency

The optical transparency of air filter/membranes is another important parameter used to evaluate their performance. When using an air filter in windows or face masks, transparency helps in maintaining visible light transmission along with good PM removal efficiency. The optical transparency is usually measured with the help of a UV–vis spectrophotometer [64]. Transmittance measurement can also be used to measure optical transmittance. PAN nanofiber is highly useful in obtaining transparent air filter. The transparency of a filter can be achieved by regulating the microstructure and surface modification chemistry. Cui et al. reported an ultrathin air filter with excellent transparency [42].

## 7. Material Scopes for PM Filtration

Emerging materials used commonly for fabrication of efficient air filters include polymers, carbon nanomaterials, porous materials, electrical filtration materials, network materials, etc. A brief overview of these materials has been given in this section.

### 7.1. Polymers

**Figure 6:**
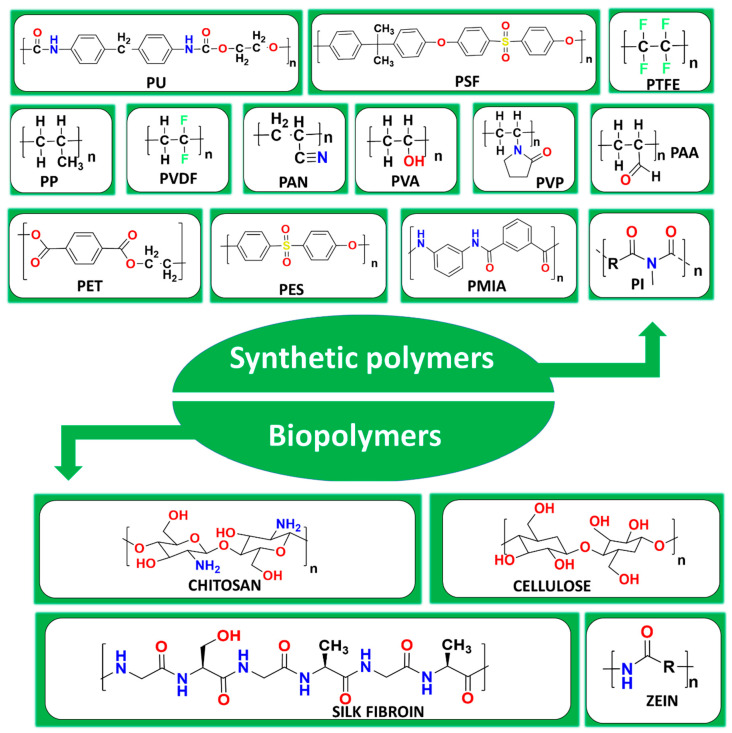
Chemical structures of common polymers used in fabrication of membrane air filters.

The wide use of polymers has been witnessed for the fabrication of air filtration membranes. In general, we can categorize polymers into two broad groups: synthetic polymers and biopolymers. In Figure 6, chemical structures of some commonly used polymers, both synthetic and biopolymers, are given. The polymers used in the fabrication of commercial air filters are polyethylene (PE) and polypropylene (PP) which are known for their affordability and ease of modification [65]. With time, a wide range of synthetic polymers have come into existence with promising applicability in air filtration. Examples of such synthetic polymers are PI [66], PAN [67], polyvinyl chloride (PVC) [68], polyvinyl alcohol (PVA) [69], polysulfone (PSF) [70], polyether sulfone (PES) [71], polyurethane (PU) [72], PVP [73], polyvinylidene fluoride (PVDF) [74], polyacrylic acid (PAA) [75], and polylactic acid (PLA) [76,77]. Electrospinning (ES) is a widely used fabrication technique for producing polymer-based NF air filters. On the other hand, the use of biopolymers such as silk fibroin [78], chitosan [79], cellulose [55,80], zein [81], and soy protein isolate [82] have also been widely witnessed in air filter fabrication for PM removal. Biopolymers are considered as very attractive materials for air filters due to their properties like biocompatibility, eco-friendliness, and biodegradability. However, some factors still limit the widespread use of biopolymers, including their susceptibility to moisture, leading to deterioration of air filters and performance loss [82]. Many attempts have been made to overcome this challenges associated with biopolymers, among which their blending with synthetic polymers and other filler materials/nanomaterials are common. This helps in enhancing the moisture resistance capacity as well as mechanical strength of biopolymer-based filters. Table 3 represents some of the performance achieved through the utilization of synthetic polymer- as well as biopolymer-based air filters.

### 7.2. Other Novel Materials

#### 7.2.1. Porous Materials

Porous materials hold tremendous scopes in the removal of PM components due to their attractive features of ultrahigh porosity, high thermal as well as chemical stability, customizable pore size and ease of functional modification [95]. Among porous materials, metal-organic frameworks (MOFs) are extensively utilized in air filtration due to their porous structure. MOFs are generally constructed from metal ions as node and organic ligands as linker, which are connected via coordination bonding [96]. MOFs are usually applied on supports like polymer NF films, cotton, or graphene aerogels, etc., which are used for PM filtration. Such examples include zeolitic imidazolate framework (ZIF) coating on a PU substrate [97], coating of UiO-67 or MIL-101 MOFs on cotton substrates [98,99] and coating of ZIF-67 on reduced graphene oxide aerogel (rGA) substrates [100].

#### 7.2.2. Carbon-Based Materials

Carbon-based materials have advantageous properties like high specific surface area, high porosity and enormous active sites for adsorption [7,101]. Among various carbon-based nanomaterials, graphene oxide (GO) and carbon nanotube (CNT) have exhibited immense potential in PM separation applications. These materials require an appropriate substrate to form an effective air filter. In situ synthesis of 1D CNT over a membrane substrate of silicone carbide (SiC) has been reported where the spiral CNT-coated membrane shows very good PM filtration efficiency of 99.48% [102]. Another study reported the use of a charged graphene aerogel filter (CGAF) for PM_2.5_ removal with a filtration efficiency of >99.6% (oily PM_2.5_) and >99.9% (non-oily PM_2.5_) [54]. A PVDF NF membrane filter functionalized with GO was fabricated via a blending-ES method and displayed high PM_2.5_ filtration performance along with good reusability and mechanical stability [103].

#### 7.2.3. Electrical Filtration Materials

Electrostatic precipitation is one of the widely used techniques for PM filtration due to its low pressure drop as well as effective purification [104]. The electrostatic effect in this procedure is highly dependent on the applied voltage. An electrostatic effect-driven PM separation filter was reported by Ko et al. where voltage was applied on an Ag nanowire membrane [105]. This filter exhibited a long-term electrostatic force and, under the influence of low voltage, showed a temporary van der Waals force [20]. Another study reported a smart window composed of Ag-nylon, which showed good PM_2.5_ filtration efficiency when subjected to a voltage of 10 V [106]. Similarly, a wide range of electric filters have been reported as effective for PM separation [107].

#### 7.2.4. Nanowire Networks

**Figure 7:**
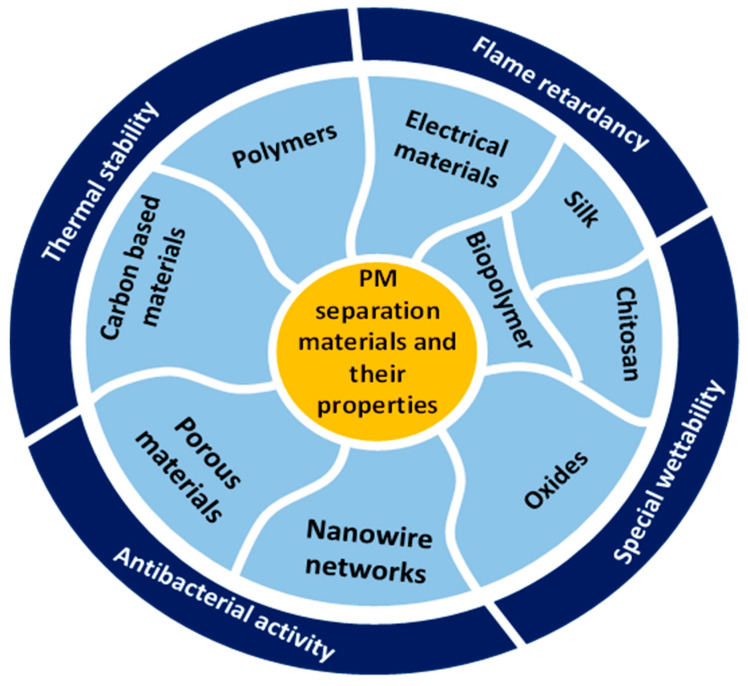
Scopes of filtration materials and their special properties for PM capture.

Nanowire networks have garnered extensive usage in optoelectronics as flexible, transparent conductors due to their exceptional chemical stability against oxidation and superior electrical, optical and mechanical properties [108]. Nanowires have emerged as promising candidates for PM filters due to their exceptional flexibility, substantial specific surface area, and intricate 3D porous network structure [20]. Example of nanowire network filters documented in the literature include Ag nanowire-nylon mesh filter [106], HAP nanowire-cotton fiber [109], etc. These filters have demonstrated significant utility in PM filtration, attributable to their high filtration efficiency, transparency, electrical conductivity, antibacterial properties, and reusability. Figure 7 demonstrates various special properties of different air filter materials. In Table 4, advantages and disadvantages of different air filter materials are listed.

## 8. Membranes for PM Separation

**Figure 8:**
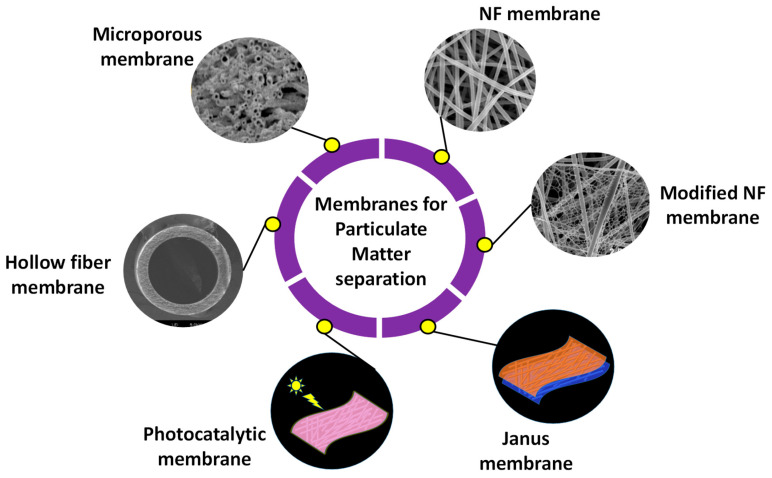
Different types of membranes used in PM separation.

Different types of membranes utilized for PM separation are discussed in this section and depicted in Figure 8.

### 8.1. NF Membranes

**Figure 9:**
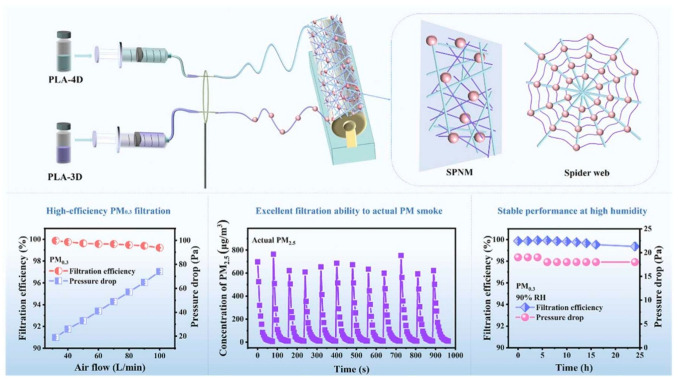
One-step fabrication of PLA NF membranes with spider-web-like structure and their PM filtration performance. Reproduced with permission from ref. [132]. Copyright © 2024, Elsevier.

NF membranes were employed for the first time in the 1980s for air filtration applications [116]. NF membranes have fiber diameter ranges from 40 to 2000 nm, which makes them appropriate for using in the separation of PM components of varied sizes [117]. Fabrication of NF membrane involves four primary techniques, viz.: multi-component fiber spinning [118], ES [119], electrospinning/netting (ESN) [60], and modular melt-blown techniques [120]. Of these methods, ES is found as the widely used one. The ES method involves a polymer solution that is placed in a capillary tube attached with a syringe needle. A high voltage is applied to the needle tip, which produces an electrically charged jet of the polymer solution. The electrically charged polymer solution is then sprayed over a metallic surface to form the desired NF membrane. ES offers significant advantages, allowing the customization of fiber diameter through adjustable operating parameters. These parameters are voltage, humidity, distance between needle and collector, viscosity, surface tension, conductivity and flow rate of the polymer solution [121]. The desire to achieve defect-free or adjustable filters with low pressure drop and high separation efficiency has led to some hybrid membranes like multilayer NF membranes [122], composite NF membrane [123] and electret NF membrane [124]. The solution blow spinning (SBS) process is also utilized to fabricate air filtration membranes. A study reported the fabrication of a PLA NF air filter on a coconut mesh via the SBS technique, which achieved filtration efficiency of 70% with a pressure drop < 60 Pa for particles having sizes in the range of 0.02–0.2 μm [77]. Several studies have reported the use of bare NF membranes for PM filtration where the membrane is made up of pristine polymers without additives [125]. The air filtration performance of NF membranes is very much dependent on the type of polymer, membrane weight, packing density, fiber diameter and fiber distribution [126]. Although higher membrane weight enhances filtration efficiency, it often presents a large pressure drop. Hence, the development of ideal air filters that are lightweight with high filtration efficiency and minimal pressure drop is needed [126]. These characteristics collectively contribute to maximize the effectiveness and practicality of air filtration systems. Aramid NF membranes possess high thermal as well as solvent resistance [127]. Reportedly, as compared to other polymeric NF membranes, meta-aramid air filters made up of poly (m-phenylene isophthalamide) (PMIA) show very high filtration efficiency and QF along with low basic weight and high mechanical strength. As measures to increase the PMIA solubility as well as spinnability, the addition of salts like lithium and calcium chloride to the solvent has been reported [128]. The use of biopolymers has also been extensively studied recently in the fabrication of NF membranes for air filtration. Some of the most commonly used biopolymers are cellulose [129,130], PLA [131,132], chitosan [133,134], zein [134], etc. Table 5 shows different NF membranes with their physical properties and filtration efficiencies. Figure 9 illustrates the fabrication of PLA-based NF membranes having a spider-web-like structure along with their PM filtration performance.

### 8.2. Surface-Modified NF Membranes

**Figure 10:**
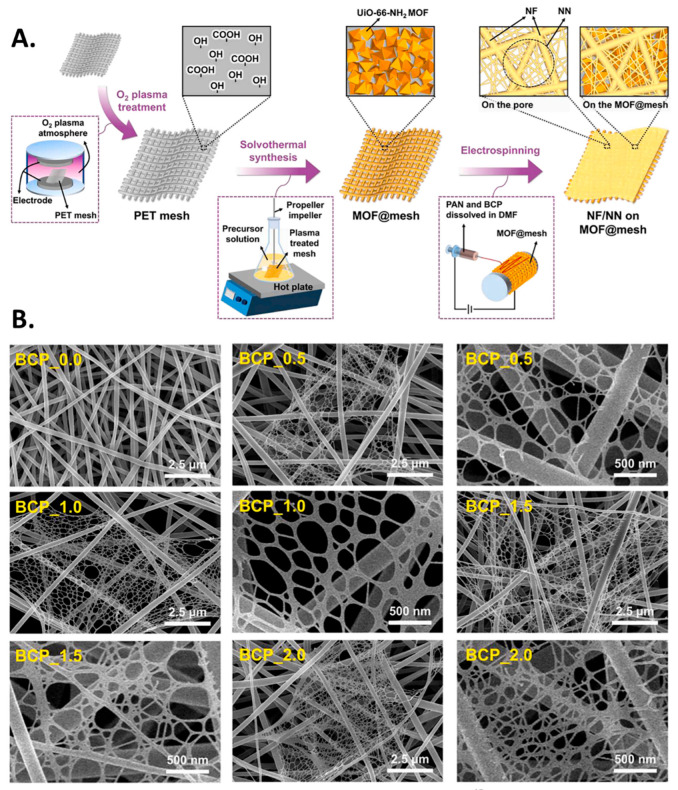
(**A**) Schematic diagram of NF/NN membrane fabrication with a MOF@mesh, (**B**) SEM images of BCP-added PAN-based NF/NN membranes fabricated with different BCP concentrations. Reproduced with permission from ref. [144]. Copyright © 2023, Elsevier.

To enhance the functionality of NF membranes, additives can be incorporated into pure polymeric air filtration membranes through functionalization or surface modification. These kinds of modifications allow the filters to separate harmful materials, something that is difficult to accomplish with pristine polymeric NF membranes. Modified NF membranes can be prepared by incorporating additives during or after the preparation process. Nano-additives like SiO_2_ nanoparticles and modified SiO_2_ nanoparticles have been extensively used in air filters made of PAN [126] and PVDF [56] polymers, respectively. Chelating agents like EDA have been used to fabricate EDA-modified MgTPP-coated PEI NF membranes for CO_2_ adsorption and PM_2.5_ capture [57]. Surface modification is an effective strategy to improve the air filtration performance of NF membranes. Such an example of surface modification is MOF-modified NF membrane, which shows impressive PM removal efficiency of 99.99% [140]. However, modification of the NF membrane surface with inorganic material is very challenging, which may be due to the lack of surface functional groups for effective interaction. Pre-functionalization of the membrane surface or inorganic material is a possible solution to overcome this challenge. Coupling agents are sometimes used to chemically bind inorganic materials to the surface of NF membranes; this not only enhances the membranes’ performance but also extends their longevity. Examples of silane coupling agents include 3-aminopropyltriethoxysilane (APTES), N-(3-(trimethoxysilyl)propyl) diethylenetriamine (TMSPDETA), and N-(β-aminoethyl)-γ-aminopropylmethyl dimethoxysilane (AEAPMDMS). These agents have been used to functionalize mesoporous materials for the adsorption of formaldehyde from polluted air [141]. Other nanoparticles commonly used in NF membrane modification are ZIF, hydroxyapatite (HAP) nanoparticle [142], metal and metal oxide nanoparticles such as Ti, CaO, MgO, Al_2_O_3_, Ni, etc. [143]. Lee et al. have designed a membrane showing both calorimetric sensing ability as well as good PM_2.5_ filtration ability [144]. This colorimetric nanofiber/nanonet (NF/NN) membrane is constructed via coating of MOFs on a mesh substrate (Figure 10A). The fabrication of NF/NN membrane involves an ESN technique where a spider web-like NN structure in between NFs is obtained after the addition of halochromic dye (Figure 10B). This membrane not only exhibits excellent sensing performance for ammonia gas but also demonstrates high filtration efficiency for aerosol particles smaller than 120 nm, achieving a QF of 0.0387 Pa^−1^. The incorporation of carbon nanomaterials like GO [103], metal nanoparticle-embedded GO [145], doped carbon dots [130], etc., in air filters has also been reported for PM removal. The fabrication of temperature-resistant air filters is both challenging and crucial. One successful approach involved creating a composite NF membrane using PMIA and TiO_2_. This composite membrane offers high efficiency in removing PM_2.5_ (filtration efficiency of 99.3% for PM_2.5_ and a pressure drop of 61 Pa) and demonstrates excellent stability at elevated temperatures (up to 250 °C) [146]. Incorporating advanced properties such as antimicrobial activity, thermal stability, photocatalytic activity, and improved wettability into air filters through surface modification have been extensively researched. Table 6 illustrates different fabrication techniques, physical properties and filtration performances of modified NF membranes.

### 8.3. Microporous Membranes

Microporous membranes function like a sieve and separate particulates based on their sizes. To attain a high filtration efficiency for PM components of various sizes, the pores of a membrane need to be tuned specifically. However, with the decrease in pore size of the membrane in order to attain high PM removal, the pressure drop tends to increase, and as a result, clogging issues arise. Conjugated microporous polymers (CMPs) are widely used for the synthesis of microporous air filtration membranes. CMPs represents a class of porous organic polymers with the advantages of large surface area, easy functionalization and tunable pore properties [148]. Moreover, the excellent adsorption capacities of CMPs for particulates makes them potential materials for PM separation. Many of the recent studies showcase the PM_2.5_ capturing ability of CMPs [149,150]. CMP-based filters are fabricated with monomers such as 1,4-dibromobenzene, 1,3,5-triacetylenebenzene, and 4,4′-dibromodiphenyl via S–H cross-coupling reactions under high- or dry-temperature environments [148,151]. These membrane air filters exhibit excellent PM_2.5_ and PM_10_ filtration efficiency even in humid conditions. CMP nanosheets are also found to be effective in the capture of VOCs [152]. CMPs in nanosheet form offer extensive surface areas which enhance their ability to adsorb different pollutants. Wang et al. reported a self-supporting CMP membrane via S–H coupling which exhibits excellent PM filtration efficiency of 99.7% for PM_2.5_ and 99.9% for PM_10_ [84]. Another attempt has been made to synthesize a hamburger-structured CMP membrane via S–H coupling reaction through a simple face-to-face constrained growth process. With a unique structure, this membrane is able to attain a very high filtration efficiency of >99.5% for PM_2.5_ and PM_10_ and >95% for PM_0.3_, where the origin of PM is engine exhaust gas [153]. The effectiveness of this membrane was also tested under highly humid conditions. Additionally, due to the lipophilic properties of these membranes, they offer impressive oil adsorption behavior. Table 7 shows the filtration performance of some CMP-based microporous membranes. With excellent pollutant adsorption ability, CMP nanotubes are also gaining lots of popularity in PM separation [150,154,155,156,157]. CMP-based microporous membranes hold significant potential for large-scale manufacturing in various industrial applications. Moreover, the integration of CMPs with other advanced materials like carbon nanomaterials, inorganic nanomaterials, etc., can be studied further to achieve higher performance and thermal, mechanical and microbial resistance.

### 8.4. Hollow Fiber Membranes (HFMs)

Compared to flat sheet membranes, HFMs provide numerous advantages, including a self-supporting structure and high packing density [158]. In an HFM module, the strategic arrangement of the straw-like fibers enables an extensive filtration surface area. Flexible HFMs of diverse morphologies and geometries can be produced by adjusting fabrication parameters. With these superior characteristics, HFMs find applications in various fields such as water treatment, gas separation, and osmotic pressure generation [159,160,161]. In the field of air filtration, HFMs reportedly showed excellent performance. Due to the porous and fibrous structure of HFMs, the separation of PM_2.5_ particles becomes easier. A PES-based HFM that was subjected to PM separation not only exhibited an impressive filtration efficiency but also provided good cleaning ability as well as longevity [162]. In the generation of HFMs, a variety of polymers have been explored, including polytetrafluoroethylene (PTFE) [163,164], PP [165], PES [162], etc. For instance, a study demonstrated the use of silver-loaded zirconium phosphate (AgZrP) in the fabrication of PTFE-based HFMs. PTFE HFMs incorporating 1 wt.% AgZrP achieved particle filtration efficiencies of 97.7324% for 0.3 μm particles and 99.9984% for 2.5 μm particles [164]. The PM separation performance of some reported HFMs is given in Table 8. Although HFM-based PM separation has not been explored extensively, this field holds enormous potential for research in the near future with the incorporation of various adsorbing materials on the surface or in the matrix of HFMs.

### 8.5. Janus Membranes

**Figure 11:**
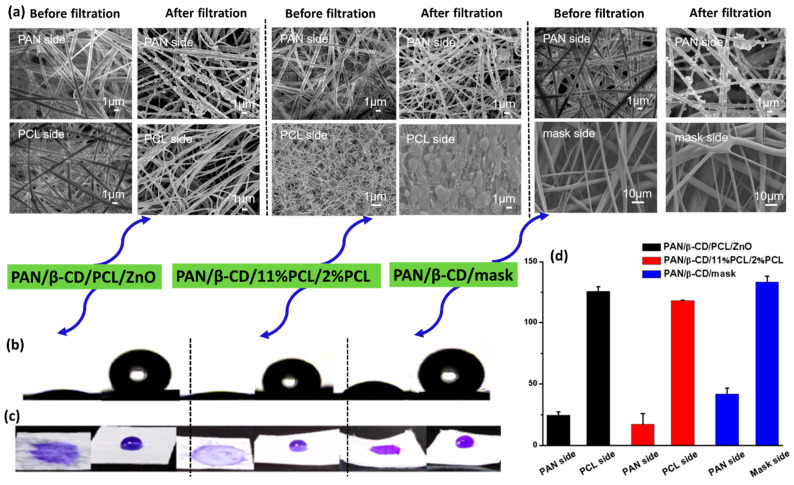
(**a**) SEM images of PAN/β-CD/PCL/ZnO, PAN/β-CD/11%PCL/2%PCL and PAN/β-CD/mask before and after 4 h of PM filtration, (**b**) optical images of water contact angle, (**c**) photograph of surface wettability and (**d**) contact angle quantitative data of the membranes. Reproduced with permission from ref. [166]. Copyright © 2021, American Chemical Society.

A Janus membrane is an advanced membrane with the property of dual wettability [166]. This type of membrane consist of one inner hydrophilic surface and one outer hydrophobic layer with self-cleaning abilities [167]. This membrane is highly applicable in addressing the common issue of fouling. The hydrophobic outer layer of a Janus membrane acts as a shield against contaminants, which not only minimizes fouling but also provides effective cleaning. Moreover, Janus membranes offer excellent chemical stability [158]. These advantageous properties make them highly efficient for applications in personal protection materials, water treatment, air filtration, etc. Cui et al. have designed a Janus membrane composed of PAN@TiO_2_/poly(vinylidene fluoride-co-hexafluoropropylene) (PVDF-HFP)@SiO_2_ nanofibers (PTPSNF) via the ES technique. The membrane demonstrates a PM_0.3_ filtration efficiency of 99.7% with a pressure drop of 27 Pa. The PVDF-HFP@SiO_2_ layer of the membrane is hydrophobic and responsible for its self-cleaning ability, while the PAN@TiO_2_ surface is hygroscopic in nature [167]. Another Janus membrane features β-cyclodextrin(β-CD)/PAN on the hydrophilic side, combined with various hydrophobic constituents, including polycaprolactone (PCL)/hydrophobic zinc oxide (ZnO), different PCL formulations, and a hydrophobic layer extracted from a commercial mask [166]. This Janus NF porous membrane offers separation of PM components of varied sizes along with effective VOC adsorption. The adsorption of pollutants by these prepared membranes is well verified with the SEM images given in Figure 11. A few studies have exploited Janus membranes as an efficient solution for air filtration as well as PM separation [168,169]. One such example is the one-pot fabrication of Janus membranes, which involves the layer-by-layer crystallization and quenching of 12-hydroxystearic acid and halicin on a PP non-woven fabric, and they are laminated with hydrophilic cotton fibers. In comparison to commercial N95 masks, these composite Janus membranes exhibit superior filtering effects, low filtration resistance (57 Pa), a QF of up to 0.072 Pa^−1^, and a contact angle of 157.1°/0° on both their superhydrophobic and superhydrophilic sides [170]. Reported PM filtration performance values along with the surface wettability of Janus membranes are listed in Table 9.

### 8.6. Photocatalytic Membranes

Photocatalytic membranes are examples of next-generation membranes which nowadays have been gaining enormous attention in PM separation due to their ability to remove particulates of submicron level. In a recent study, a light-driven air filter membrane was designed based on Schottky junction photocatalysts and was found to be highly applicable. In this Schottky junction, the free flow of electrons from the semiconductor to the metal generates a built-in electrostatic field which facilitates the PM separation [171]. Another study reported the fabrication of a photocatalytic membrane with a TiO_2_/g-C_3_N_4_ heterojunction photocatalyst coated on a PS/PAN multiscale NF support. The membrane was fabricated by a very simple one-step blending process followed by the ES technique. The built-in electric field, characterized by strong long-range electrostatic forces, enhances filtration efficiency under light. Under challenging conditions of high flow rate (25 L/min) and high PM concentration (>1,800,000 m^3^), this membrane achieved a PM filtration efficiency of 98.5% with a pressure drop of 94 Pa [172]. The use of photocatalytic membranes in PM separation is still an emerging field, and these membranes have great potential for further development. With the exploration of various photocatalysts, one can finely tune PM separation efficiency and other properties of photocatalytic membranes.

## 9. Critical Comparison of Cost, Durability and Scalability of Different Types of Membranes

A comparative analysis of different types of membranes in terms of cost, durability and scalability is very important to understand their feasibility in real-time PM separation applications. First of all, NF and microporous membranes should be compared in this respect. Electrospinning is one of the widely used technique to fabricate NF membranes which greatly depends on the polymer solution, processing parameters and fabrication environment. NF membranes possess exceptionally high surface area, interconnected pores, high permeability, and controlled selectivity as well as easy functional tunability [173]. Reportedly, BCC research, global markets, and technologies for nanofibers have predicted the global market for nanofibers would increase from USD 927 million in 2018 to USD 4.3 billion by 2023 with a compound annual growth rate of 36.2%. Although both the solution method and melting method are used in ES technology, the application of the solution method is mostly preferred. The solution electrospinning method offers cost-effective and smooth operation, making its use in the laboratory as well as industry highly feasible [173]. However, one of the major disadvantages is the requirement for very high voltage during the ES fabrication process. Mechanical stability is also not very high, and post-modification strategy like cross-linking or reinforcement is often required. Incorporation of filler materials like polymers or inorganic nanomaterials is commonly applied to enhance the mechanical properties of electrospun nanofiber membranes [174]. Optimization of process parameters during electrospinning-based membrane fabrication also improves nanofiber quality. On the other hand, microporous membranes can be fabricated by various cost-effective and scalable fabrication methods like phase inversion, stretching, sintering, etc. [175]. However, relatively lower permeability and susceptibility to fouling often make the microporous membranes disadvantageous. NF membranes have become more advantageous by virtue of their high permeability and functional tunability, whereas microporous membranes are more suitable for large-scale usage and industrial applications. Interestingly, HFMs emerged as a promising choice of membrane providing high surface area, compact size, self-supporting characteristics and efficient PM separation performance. In terms of scalability and cost-effectiveness, HFMs demonstrate significant superiority over other membranes. HFMs also provide simple fabrication, easy scaling up, flexible modulation, and regenerability, which makes them highly feasible in industrial applications [176]. When using HFMs, the cross-flow mode is often used, which minimizes the tendency toward fouling and enhances the life span of the membrane. However, limitations such as plasticization, fragility and flux versus selectivity trade-offs are observed in HFMs. Janus and photocatalytic membranes are often composed of various advanced materials, which make them costlier than the conventional membranes [177]. However, in terms of durability, these membranes withstand repetitive cycles and retain their performance for longer periods of time via their antifouling and self-cleaning ability.

## 10. Important Functionality of Membranes for Air Filtration

### 10.1. Antibacterial Activity

**Figure 12:**
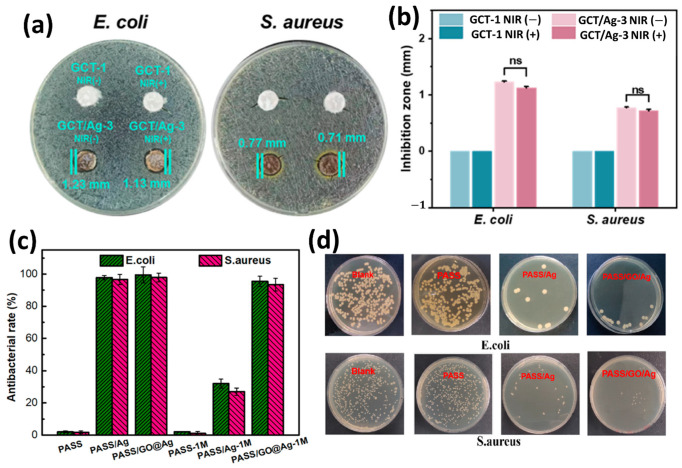
(**a**,**b**) Antimicrobial activities and inhibition zone of GCT/Ag air filter against *E. coli* and *S. aureus*. Reproduced with permission from ref. [21]. Copyright © 2024, Elsevier. (**c**) Characterization of antibacterial properties of different PASS nanofiber membranes. (**d**) Antibacterial performance of PASS, PASS/Ag, PASS/GO@Ag, PASS-1 M, PASS/Ag-1 M, and PASS/GO@Ag-1 M against *E. coli* and *S. aureus.* Reproduced with permission from ref. [145]. Copyright © 2023, Elsevier.

One of the important properties of membrane air filters is antibacterial activity. PM components are aerosols that often carry bacteria and viruses, which, upon exposure, lead to various diseases. Antimicrobial properties of membrane air filters can inhibit the growth of harmful bacteria, viruses, etc. This not only reduces the health issues related to airborne pathogens but also helps in maintaining consistent performance and extends the lifetime of the membrane. The inclusion of antibacterial property in membrane air filters minimizes the need for their frequent replacement and maintenance costs [178]. Among different materials such as cotton, non-woven fabrics, melt-blown materials, and electrospun fibers, ES NF membranes are found to be the most effective in air filtration [179]. Currently, many studies have focused on incorporating inorganic nanoparticles into membranes to introduce antibacterial properties. Commonly used inorganic nanoparticles include Ag NPs [180], titanium dioxide particles [181], copper NPs [182], zinc oxide particles [183], quaternary ammonium salts [184], N-halamine compounds [185], and *Garcinia mangostana* L. [186], GO nanosheet [180]. Amongst these, Ag NPs are the most widely used in the fabrication of various antibacterial air filters like the GCT/Ag air filter [187] and PASS/Ag-based air filter [145]. The antimicrobial performance of these filters is shown in Figure 12. Although inorganic nanoparticles show excellent antimicrobial properties, they are often found to be costly in nature, toxic and susceptible to degradation [188]. That is why research has shifted towards bio-based antibacterial agents. Choi et al. used Sophora flavescens plant extract as an antimicrobial agent in PVP-based electrospun NF membranes. This membrane shows remarkable antimicrobial activity of 99.98% against *Staphylococcus epidermidis* [189]. Another study reported the use of cinnamon oil, which is known for its antibacterial properties, for designing AC and PU polymer-based air filter membranes. These cinnamon oil-incorporated filters show strong resistance against *Staphylococcus aureus* (*S. aureus*) and *Escherichia coli* (*E. coli*) [190]. Berberine is another naturally available alkaloid which has been used in traditional Chinese medicine due to its antibacterial properties. Reportedly, berberine is utilized in the fabrication of antibacterial membrane filters [191]. Other effective antibacterial agents are Ag NPs, titanium oxide NPs, etc., which have found application in the design of antibacterial NF membranes [192]. Table 10 lists antibacterial performance of a few reported air filtration membranes.

### 10.2. Wettability

The wettability of an air filter membrane significantly impacts filtration performance, self-cleaning capabilities and reusability. Numerous studies have explored the influence of wettability on filtration performance [194,195]. In highly humid conditions, to attain good filtration performance, hydrophobic filtration materials are preferred. Otherwise, moisture tends to condense and block the filter while increasing the pressure drop. Hydrophobic filters can reduce this type of blocking-induced pressure drop and can release dust from the membrane surface while making the membrane durable. For instance, a hierarchical air filter with water-repellent properties has been developed by growing CNTs on quartz fibers, achieving a water contact angle of 143° [195]. An electret air filter prepared from polyvinyl butyral (PVB) and fluorinated PU (Si_3_N_4_-FPU) has been reported to exhibit exceptional charge stability [196]. Another hydrophilic electrospun fibrous membrane with PAN and SiO_2_ has been reported to demonstrate efficient PM capture ability along with enhanced moisture transfer [197]. A superoleophobic filter coated with fluoropolymer has been reported with superior filtration capability [198].

### 10.3. Thermal Stability

**Figure 13:**
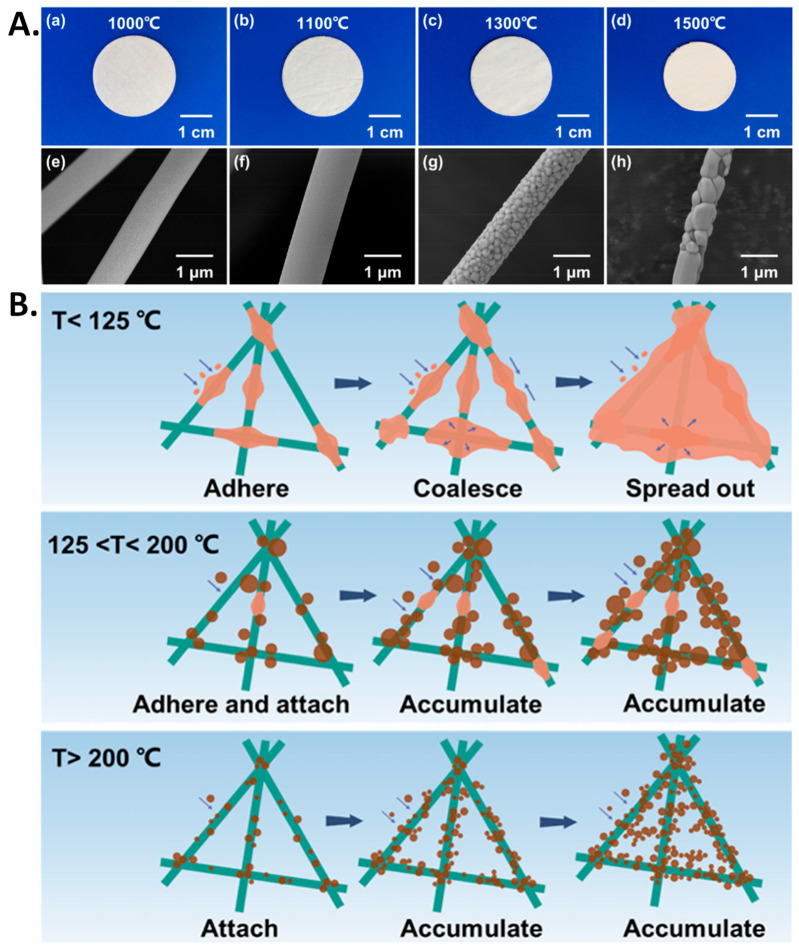
(**A**) (**a**–**d**) Optical images and (**e**–**h**) SEM images of the ASZ papers produced at different treatment temperatures. Reproduced with permission from ref. [203]. Copyright © 2020, American Chemical Society. (**B**) Schematic illustration showing the different processes of PM capture by Zr_0.15_Ti NF membranes at 25–350 °C. Reproduced with permission from ref. [204]. Copyright © 2023, Elsevier.

Industrial processes generate exhaust gases at high temperature. The exhaust gas is often contaminated with PM that contributes significantly to air pollution [20]. For separation of PM components present in high-temperature exhaust gas, high temperature-resistant air filters are needed. PIs are widely used in such applications due to their excellent thermal, chemical and mechanical stability. Cui et al. introduced a PI NF air filter with exceptional thermal stability and outstanding PM filtration [199]. Up to a temperature of 370 °C, PM_2.5_ filtration efficiency of the membrane was found to remain unchanged. In extreme heat also, 99.5% separation of car exhaust-generated PM was achieved. SiO_2_ NF membranes are widely used in air filtration due to their excellent thermal resistance and versatile performance. Such SiO_2_ NF membranes have been produced through ES and sol-gel processes, which have been found to maintain both their efficiency and structural integrity even at temperatures reaching 1000 °C [200]. Alumina is another example of an inorganic material that has found application in PM separation due to its high melting point and chemical and thermal resistance. A γ-alumina NF film was reported to exhibit excellent thermal resistance up to 900 °C, and under calcination at 700 °C, achieved PM filtration efficiency of 99.848% with a low pressure drop of 239.12 Pa [201]. A thermally oxidized SnO_2_-based PAN/PVP NF membrane has been reported with excellent PM filtration efficiency of 98.51% (at 350 °C) and 98.67% (at 300 °C) [202]. A flexible air filter made of Al_2_O_3_-stabilized ZrO_2_ (ASZ) submicron fiber was reported to show high air filtration efficiency with high temperature resistance up to 1100 °C [203]. After an optimum temperature of 1100 °C, a morphological change in ASZ air filter occurred, confirmed by optical and SEM images at different temperatures (Figure 13A). Zr-doped TiO_2_ NF membranes were reported to show good PM filtration efficiency at 350 °C (Figure 13B) [204]. With further investigation on temperature-resistant materials, durability as well as the performance of membrane air filters can be enhanced even in extreme temperature environments.

### 10.4. Flame Retardancy

**Figure 14:**
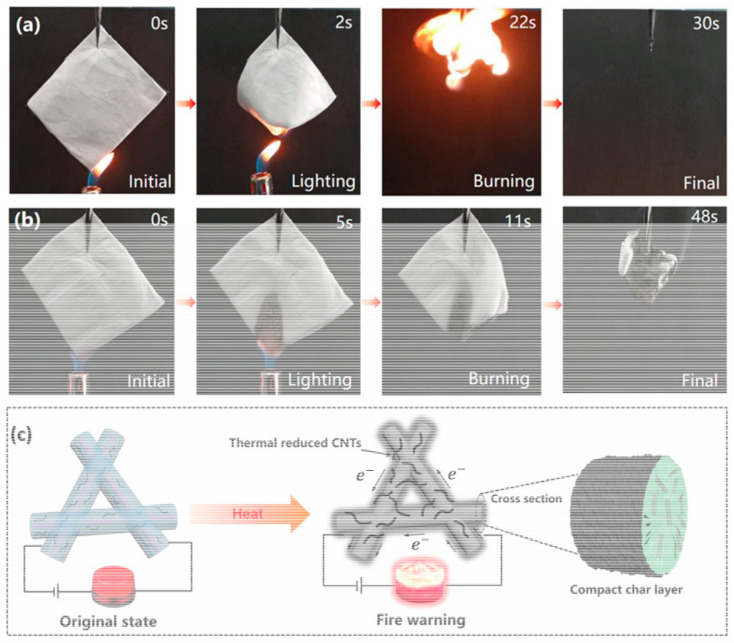
Burning test for (**a**) pure PAN and (**b**) PAN/CNTs/APP fiber filters. (**c**) Fire-warning mechanism of PAN/CNTs/APP filters. Reproduced with permission from ref. [206]. Copyright © 2022, Elsevier.

One of the important properties that needs to be critically considered is flame retardancy of the separating media. Flame retardancy allows the air filtration media to withstand extreme conditions, including exposure to fire and high temperature conditions. This property is particularly crucial for designing air filters to withstand fire- or extreme heat-prone environments, such as industrial facilities, power plants and transportation systems. By incorporating flame-retardant materials, the structural integrity and performance of air filters can be maintained under challenging conditions. A flame-retardant NF membrane prepared with tri-phenyl phosphate@Nylon-6 showed very good PM filtration efficiency [205]. The neat Nylon-6 membrane tends to combust under fire exposure, while the tri-phenyl phosphate@Nylon-6 membrane under ignition shows self-extinguishing capability. Thus, tri-phenyl phosphate can be considered as a very good material of choice for use in flame-resistant air filters. The combination of PAN/PVP/SnO_2_ to prepare an NF membrane for PM filtration is an excellent example of a material with fire resistivity and self-extinguishing properties [202]. Another study reported a filter with PAN which was modified with ammonium polyphosphate (APP) and amino-functionalized CNTs (A-CNTs) [206]. The PAN/APP/A-CNT filter possesses both flame retardancy as well as fire warning abilities. A vertical burning test conducted for this filter (shown in Figure 14a,b) found that the fire warning response time was ~5 s. The A-CNTs were converted to reduced A-CNTs while burning and formed a char layer. As a result, the conductivity of the filter was enhanced and, in turn, the fire warning behavior was obtained (Figure 14c).

### 10.5. Reusability

Reusability is one of the important properties of an air filter to achieve both economic as well as environmental sustainability. Reusable air filters offer a cost-effective route for PM separation while minimizing the need for frequent replacements. A triboelectric filter made from nylon and PTFE has been developed, capable of being washed with both water and detergent [107]. Remarkably, this filter maintains a filtration efficiency of 92% even after being washed five times, whereas commercial filters typically show a reduction to 67% in filtration efficiency. An Ag nanowire-containing air filter reported with notable reusability is washable with ethylene glycol multiple times without loss of mechanical strength, electrical resistance or PM separation performance [105]. Another washable filter, made of hierarchical ZIF-L (H-ZIF-L)-modified PP polymer, was reported to offer multiple washings of 12 cycles with retention of its original PM_10_ filtration efficiency [88]. A super amphiphobic reusable NF membrane has been reported with PM_2.5_ filtration efficiency of 99.9% and is recyclable up to 5 times when washed with water [207]. However, a few drawbacks still remain, such as maintenance and a time-consuming drying process after washing. Other issues like a decrease in filtration efficiency and increased pressure drop also arise in air filters after multiple washes.

Fouling is a major challenge in membrane-based separation procedures. Rejected PM deposits on the membrane pores, thereby diminishing the membrane’s performance. Various strategies have been implemented to reduce this fouling. The most commonly used procedure is hydrophilic functionalization on the membrane [208]. For example, a study by Sun et al. discussed broadly how hydrophilic modification helps membranes in attaining antifouling properties [208]. Most of the techniques used for hydrophilic modification of membranes involve plasma modification, surface coating, irradiation grafting and blend modification. Hydrophilic modification can introduce a hydration layer on the membrane surface which acts as a barrier and prevents the adhesion of PM on the membrane surface and pores. Another important modification is incorporation of nanoparticles in the membrane during fabrication, which imparts antifouling and antibiofouling behavior. Nanomaterials like graphene, its derivatives, carbon nanotubes, g-C_3_N_4_, layered double hydroxides, carbon dots, and activated carbon have shown very good antifouling behavior [209]. Carbon nanomaterials exhibit excellent antibacterial properties by damaging cell walls of bacterial pathogens and thereby preventing bacterial activity and proliferation. The inclusion of photocatalytic properties in membranes is another innovative modification for effective and long-term PM removal. Photocatalysts can degrade the deposited PM components on the membrane under the influence of UV–visible irradiation, which opens the blocked pores and provides the membrane a longer lifetime with excellent performance [210]. In this way the membrane’s life time for long-term performance can be enhanced significantly.

## 11. Commercial Air Filters for PM Separation: Availability and Challenges

Air filtration through membrane-based methods has been widely used to ensure good indoor air quality by capturing airborne pathogens, which is helpful in preventing asthma and maintaining air sterility in controlled environments. Modern air filtration technology comprises diverse systems and filter media and is often integrated into HVAC systems, air conditioners, and air purifiers. These systems typically involve multiple filtration steps and are usually designed by using inexpensive and disposable materials like glass or synthetic fibers. Commercial air filters play a significant role in maintaining the indoor air quality across various localities. These filters are mainly employed in residential homes in metropolitan areas where air pollution levels are high.

At present, commercial air filters are in high demand and are used in cities with elevated pollution levels. Amongst various commercial air filters, HEPA filters can be considered to be one of the most effective filters. HEPA filters physically strain out PM components from the air. They are highly efficient for particles of 0.3 μm in size. HEPA filters can capture 99.97% of particles smaller than 0.3 microns, making them essential for applications requiring stringent air purity standards, such as hospitals and clean rooms. Chemfit India has developed HEPA filters which can separate PM_2.5_. The commercial names are Mini Pleat HEPA Filter–H13 and H14. Particles smaller than 0.3 μm, including bacteria or unattached viruses, are also equally or more efficiently captured by HEPA filters via interception and diffusion mechanisms. MERV 13 filters, another efficient air-filter type, are always recommended for infection control but are less efficient than HEPA filters for removal of sub-micron particulates. Recent study shows that MERV 13 filters can separate 50–80% of particles of 0.3–1 μm in size. After COVID-19, MERV 13 filters have been recommended in many commercial buildings as the minimum recommended standard to reduce airborne infectious aerosols. Mechanical filters like HEPA and MERV 13 cannot capture gaseous pollutants like volatile organic compounds (VOCs) or other toxic gaseous pollutants. Also, in the case of MERV 13, a significant pressure drop can be observed, which creates a concern regarding major upgrades in the fan system. In recent days, many air filters have been developed by integrating them with nanofiber coatings, pleated media and V-bank configurations which have significantly improved their efficiency in the removal of PM components from polluted air. Some commercial air filters available in the market are V-bank filters (INR 5100 per piece on Tradeindia.com), Box Type V-Cell Filter HVAC (INR 4461 per piece on made-in-china.com), RS PRO Intrepid Pleated Panel Filter, Trident Industrial Air Filter T-100, 4YUX6 High-Capacity Pleated Filter, etc. The global market for commercial air filters has been expanding day by day due to growing concerns about air quality both indoors and outdoors. The global HEPA filter market has grown from USD 4.93 million in 2024 to USD 5.38 billion in 2025 and is expected to grow at a CAGR of 9.05%, i.e., reaching USD 9.88 billion by 2032. The value of the market for the MERV 13 is USD 5.14 billion and is expected to grow at a 6% CAGR, i.e., to USD 8.6 billion [211,212]. Commercial air filters are considered to be a cost-effective method for the removal of PM components from ambient air due to their ability to enhance HVAC performance, lower energy consumption and low maintenance cost. Highly efficient air filters like HEPA filters or activated carbon units have comparatively high cost; however, operational cost is much lower in these commercial air filters. The life span of commercial HEPA filters is good, and they can be used for a maximum of 2 years. Industrial-grade filters have a maximum life span of 3 years. By using air filters in industrial premises as well as residential apartments, air quality can be improved, thereby minimizing the risk of exposure to health-hazardous PM.

The commercial air filters also possess very good scalability due to their modular design, well-defined manufacturing process, and high availability of raw materials. Most of the air filters like activated carbon-based air filters, cellulose nanofiber-based air filters or pleated media-type filters have been commercialized and are produced in high volume. The growing concern for unsustainable air quality has increased demand for efficient and durable air filters. The scalable nature of commercial air filters ensures their consistent supply, thereby supporting their growing applicability.

AC filters are very effective in removing odors and VOCs through adsorption mechanisms, and are often used in combination with other filters to enhance overall air purification. One of the commercially used AC filters are the Kaeser compressed air filters, which can function up to a pressure of 16 bar. Another USA-based AC air filter is Astro-Sorb™. Astro-Sorb™ has been employed in the residential and commercial industries, and it is featured with high dust holding capacity and odor adsorption. The AC-decorated air filters work as a sponge, effectively adsorbing harmful gases and vapors and preventing them from reaching the human body. Some other commercially available air filters are MMFCNY499, QPLLAV121202G4, Bag-Glass 60, MV122412F8, etc. [213]. However, adsorption-based air filtration systems are limited in their long-term applicability due to the finite adsorption capacity of the adsorbent materials, which diminishes over time. Filters and filter bags with the sensor-based ability to detect PM emission are also extensively studied nowadays [18,214]. Compared to other conventional techniques, membrane technology has the potential to offer a sustainable and energy-efficient solution for air filtration. Even with their considerable promise, there is still room for further study on air filtration membranes. From a commercial point of view, membrane technology presents an emerging solution, as it may offer the advantageous features of a large surface area when compared to other technologies. This aids in enhancing the efficiency and scalability of the filtration system. When designing air filtration materials, some other criteria also need to be addressed. The antimicrobial activity of the separation media is a key criterion in the design of air filtration materials. This property is not emphasized in many existing commercial air filters.

## 12. Future Perspectives

With the development of emerging materials and technologies, air filtration membranes hold promise for future advancement. The invention of new materials, including nanomaterials, novel polymers or composites with superior filtration efficiency, durability, and environmental resistance led to a further revolution in air filtration membrane technology. Carbon-based materials are best known for their high thermal resistance, flame retardancy, chemical stability, extensive surface area and functionalization scopes, which make them promising candidates for use in air filtration membranes. Some carbon nanomaterials such as graphene oxide exhibit inherent antimicrobial properties which are highly beneficial for air filters. However, improvements in the large-scale production of carbon nanomaterials at low cost are very important for their sustainable future application. Another important class of material is porous materials, which include newer classes of materials such as MOFs, covalent organic frameworks (COFs) and porous aromatic frameworks (PAFs). They hold enormous potential in capturing a wide range of pollutants, from ultrafine particles to volatile organic compounds. These nanomaterials not only exhibit high surface area, excellent adsorption capacity and tunable pore size but also, sometimes, demonstrate excellent antibacterial properties, which can be considered beneficial for enhancing the effectiveness, longevity, and hygiene of air filters. Recently, a group of scientists explored the potentiality of COFs and MOFs for PM separation. Ma et al. designed a Ag-MOFs@CNF@ZIF-8 cellulose-based air filter for the removal of PM_2.5_. The designed air filter showed 94.3% separation efficiency for PM2.5 removal along with antimicrobial properties and excellent mechanical strength [96]. In another work, Zhang et al. designed a MOFs/textile composite material which efficiently captures more than 95% of PM_2.5_ and PM_10_ [215]. Lee et al. designed a photocatalytic MOF and tested it for the removal of volatile organic compounds. The designed filtration system shows high efficiency for VOCs removal with recyclability [216]. To date, porous organic frameworks have not been explored for PM removal. Thus, in the near future, applications of various engineered porous materials with tailored functionalities can be used potentially to manufacture lightweight and efficient PM separation filters/membranes. Stable immobilization of carbon nanomaterials as well as porous materials is another important criterion to ensure risk-free filtration.

Nanowire networks possess advantageous features such as high filtration efficiency, lightweight nature, transparency, mechanical robustness and functionalization scopes which make them highly applicable for PM filtration. Nanowires organized in interconnected networked form with a fiber diameter in the nano range can exhibit excellent PM capturing ability. The coupling of nanowire materials with advanced catalysts, antimicrobial materials and sensors can be attempted in the near future to introduce smart PM filtration materials. To induce multifunctionality, the incorporation of various other materials such as carbon materials and porous materials in nanowires can be pursued.

Electrical filtration materials offer highly efficient PM separation by capturing particles through electrostatic interaction. These types of materials provide a porous and breathable air filter with low pressure drop, which is a key characteristic of a good air filter. In future research, advanced electret materials can be designed to retain their surface charge for a prolonged period of time, which not only increases the durability of the filter but also minimizes its need for frequent replacement.

Nowadays, self-cleaning membranes with extended life spans and requiring only minimal maintenance are in high demand. To deliver these properties, photocatalytic membranes and Janus membranes are potential candidates but will require further exploitation in the near future. A long-lasting, reusable membrane can reduce waste generated from air filter disposal. At the same time, attention should be given to the biodegradability of air filters. While various biopolymers have been explored for air filter production, there are still many available biomaterials/biopolymers, such as alginate, sericin, cellulose, etc., which potentially may be used in air filter membranes.

For the fabrication of NF membranes, the commonly used ES technique is not environmentally benign, as it involves the use of substantial quantities of toxic organic solvents. These solvents can volatilize into the air, leading to harmful environmental and health impacts [217,218]. Additionally, ES requires the supply of high-voltage power, which can contribute to high energy usage. In addition to these drawbacks, the slower speed of fabrication may restrict large-scale production. To reduce reliance on harmful organic solvents, green alternatives such as solvent-free melt processes can be employed. To address the challenge of slow synthesis, utilizing multiple needles for electrospinning could be an effective fabrication technique. Recently, such approaches have been taken to generate a green ES method for the fabrication of NF membranes by utilizing biopolymers, non-toxic solvents, surfactants and minimal energy [219].

Another undeniable issue identified by the current studies is membrane fouling, which often results from the accumulation of PM components over the membrane surface and within its pores. Membrane fouling may lead to a significant decline in membrane performance. The trade-off between selectivity and permeability is another issue which needs to be carefully studied. Moreover, the regeneration, longevity, and scalability of membrane air filters remain significant challenges that were not addressed in this study and require further, extensive investigation. The safe and sustainable disposal of used membrane materials contaminated with hazardous PM components is another environmental issue that needs to be critically considered in future research.

In order to achieve advances in membrane technology for PM removal, research and development should shift from generalized modifications towards more disruptive and actionable research directions. In this case, artificial intelligence (AI) can be implemented in membrane design and modelling, and it will provide a pathway to accelerate the design of high-performing and error-free membranes. AI-driven modelling can be implemented in optimizing the composition of materials in air filters, the accurate prediction of air-flow dynamics, pressure drop, error estimation, etc. Machine learning algorithms can be implemented to identify and select suitable biodegradable and energy-efficient materials for the design of eco-friendly filtration systems. AI will also be helpful in finding the best combination of polymers, fillers, and nanoparticles and optimizing the structural properties to minimize fouling and increase service life. The implementation of green synthesis methods will further boost the development of environmentally friendly air filters through the use of biomaterials, green solvents and green synthesis routes. AI can be helpful in predicting the green and sustainable route for designing air filters. The utilization of waste materials in the design of air filtration systems may be another sustainable and cost-effective approach. This approach will be beneficial in improving air-quality management while reducing the environmental burden. Together, the use of AI and green synthetic procedures will facilitate the development of next-generation air filters for efficient separation of harmful PM components from residential as well as industrial premises, thereby significantly contributing to a sustainable environment.

Another important way of designing high-performing membrane is to hybridize various processes, like the coupling of membrane technology with photocatalysis, adsorption, antimicrobial functionality, etc. This procedure can provide simultaneous removal of different types of contaminants like inorganic, organic and biological contaminants in a single operation.

It is also essential to consider sustainability during membrane fabrication. Special emphasis should be given to life cycle assessment, recyclability, and energy-efficient membrane fabrication, and beyond these, scalable green fabrication strategies like organic solvent-free fabrication, the design of bio-based membranes, and the utilization of biobased materials should be focused for future research. Designing self-cleaning and self-healing membranes is another approach to mitigate membrane fouling. Research and development efforts concerning such types of membranes are underway. Together, these research directions will not only pave a way towards next-generation membrane designs but also facilitate an environmentally and economically sustainable effort to remove hazardous PM components from the atmosphere.

## 13. Conclusions

PM is a key air pollutant known for aggravating haze and posing measurable impacts on both the climate as well as the environment. PM has become a concern due its ability to carry viruses and bacteria, which may cause adverse health issues in living organisms. Therefore, to eliminate these harmful environmental and health impacts of PM, it is of utmost importance to separate it from ambient air. To address this issue, various PM filtration techniques have been employed, and among them, membrane technology emerges as one of the efficient approaches. This review article discussed the latest advancements in membrane technology for PM filtration and investigated the pros and cons of existing membrane technology. Trends in membrane materials along with their types and filtration efficiencies and various approaches to PM separation using membrane technology were explored. Key membrane functionalities, including antimicrobial properties, thermal and mechanical stability, wettability, flame retardancy and reproducibility of membrane technology, were highlighted in this review article. Additionally, this review presented the current status of commercial air filters and challenges encountered. To our knowledge, there is no comprehensive review which has addressed all these aspects in a single article. This review sought to fill this gap by providing insights into current trends and future prospects in membrane technology for PM filtration.

## Figures and Tables

**Table 1:** Standard permissible limits of PM_2.5_ and PM_10_ set by WHO, NAAQS (India) and UK (sourced from https://www.c40knowledgehub.org/s/article/WHO-Air-Quality-Guidelines? & Ambient Air Quality Monitoring in India|CPCB Guidelines & Compliance-Perfect Pollucon Services (accessed on 29 July 2025)).

Pollutant	Averaging Time	Air Quality Guideline Levels Set by WHO ^b^	Air Quality Guideline Levels Set by NAAQS ^a^	Air Quality Guideline Levels in UK
PM_2.5_	Annual	5 μg/m^3^	40 μg/m^3^	-
	24 h	15 μg/m^3^	60 μg/m^3^	-
PM_10_	Annual	15 μg/m^3^	60 μg/m^3^	-
	24 h	45 μg/m^3^	100 μg/m^3^	40 μg/m^3^

^a^ NAAQS = National Ambient Air Quality Standards, ^b^ WHO = World Health Organization.

**Table 2:** General methods of PM separation versus membrane: merits and demerits.

Methods	Separation Principle	Cost	Pollutants	Merits	Demerits	Ref.
Mechanical air filter	Size exclusion	High	PM_2.5_ PM_0.3_	Good separation efficiency	Pore clogging	[46]
HEPA	Brownian motion	High	PM_2.5_ (≤) PM_0.3_	High separation efficiency	Pore clogging, Low durability	
ESP	Electric voltage	High	PM (all)	Separates pollutants irrespective of size	Requires high voltage	
AC	Size exclusion	-	Gaseous pollutants	Offers separation of gas pollutant	Not applicable to pollutants like PM	
UV light purification	Oxidative degradation	-	Bacteria, pathogens	Simple operation	Not applicable to pollutants like PM	
NF filters	Size exclusion, adsorption	Low	PM_2.5_, PM_10_	Tunable pore size, pore density, and significant aspect ratio, cost-effective, energy efficiency, high permeability	Poor thermal and mechanical properties, pore clogging	[15]
Membranes	Size exclusion	Low	PM_2.5_, PM_10_		Poor mechanical and thermal properties, membrane fouling	
Cyclone separator	Centrifugal force	Low to moderate	PM_10_	Low maintenance, cost-effectiveness, durability, robustness, versatility	Low efficiency for fine particles, significant pressure drop, sensitivity to sticky/wet materials, etc.	[48]

**Table 3:** Common synthetic polymer- and biopolymer-based air filters and their PM_2.5_ filtration performance.

Materials	Polymers	PM Size (μm)	E_PM_ (%)	∆P (Pa)	QF (Pa^−1^)	Ref.
Synthetic polymers	PI	2.5	99.97	73	0.1072	[13]
	PS	2.5	99.99	145	0.15	
	PU	2.5	99.73	28	0.211	
	PAN	2.5	96.12	133	0.024	
	PVA	2.5	96.70	178	0.019	
	PVP	2.5	95	101	0.029	
	PAA	2.5	99.6	146.3	0.034	
	PVDF	2.5	98.16	30	0.12	
	PVA/carbon nanoparticle/tea leaf extract	10	99.29	110	-	[83]
	Self-supporting CMP membrane (SS-CMPs-M)	2.5	99.7	550	0.01337	[84]
		10	99.9	550	0.01383	
	Porous gradient geopolymer-based tube membrane (PGTM)	2.5	96.5–98.7	0.01 MPa	-	[85]
		10	98.0–99.5			
	PLA/ZnCo-ZIF fibrous membranes	2.5	90.88	-	-	[86]
		10	93			
	Holey-reduced graphene oxide membrane	1.0	98.99	-	-	[87]
		2.5	99.91			
		5	99.99			
		10	99.99			
	Zn-based zeolite imidazole frameworks (2D-ZIF-Ls)	2.5	92.5	10.5	-	[88]
		10	99.5			
Biopolymers	Silk fibroin	0.3	91.4	92	-	[89]
		0.5	95.4			
		1.0	98.3			
	Cellulose nanofibril	0.3	94.6	174.2	0.0168	[80]
	Zein	0.3	97	-	-	[81]
	Chitosan	2.5	98.3	59	-	[79]
	Cellulose	10	99.08	31–34	-	[90]
	Zr-MOF-NO_2_/cotton	10	89.5	31	185.6	[91]
	Ultrathin Al_2_O_3_ on microporous cellulose ester membranes	1	76.4	135	-	[92]
		2.5	94			
		10	95.1			
	PVA/Cellulose nanocrystal composite nanofibrous filter	2.5	99.1	91	0.052	[69]
	Chitosan/NH_2_-MIL-53	2.5	98.41	24.10	0.1718	[93]
		10	99.05			
	Chitosan/PVA–SiO_2_ nanofiber	2.5	96.94	15.7	0.05941	[94]
		10	99.34			

**Table 4:** Air filter materials: advantages and disadvantages.

Air Filter Materials	Advantages	Limitations	Ref.
Synthetic polymers	Easy processing: even at industrial scale High thermal/mechanical strength Tunable fiber diameter, porosity Long durability	5. Non-biodegradable 6. Trade-off between filtration efficiency and pressure drop 7. High energy processing	[110]
Biopolymers	Biodegradable Renewable High surface functionality: tunable surface Low cost	5. Low mechanical/thermal strength 6. Sensitivity to humidity 7. Trade-off between filtration efficiency and pressure drop	[82]
Porous materials	High surface area Well-defined porous structure High porosity High adsorption capacity Ultrahigh porosity High thermal as well as chemical stability Ease of functional modification	8. Large pressure drop due to dense packing 9. Powered MOF may clog the pipes 10. Loss of active materials during the application 11. Brittle nature, insolubility, and less compatibility of MOF with substrate hinder smooth fabrication	[95,111]
Carbon-based materials	High surface area Tunable surface chemistry High mechanical strength and chemical stability High PM adsorption capacity	5. Toxicity 6. Leaching possibility 7. Agglomeration/dispersion issue [ref]	[112,113]
Electrical filter materials	High separation efficiency for submicron-level particulates Low pressure drop	3. Sensitivity to humidity 4. Charge decay 5. Temperature dependency	[114]
Nanowire networks	Chemical stability against oxidation Superior electrical, optical and mechanical properties Transparency Electrical conductivity	5. Difficulty in scaling up 6. Leaching of nanocomponents 7. Some metal nanowires are corrosion-prone	[108,115]

**Table 5:** Fabrication technique, physical properties and filtration performance of some NF membranes.

Polymer	Fabrication Technique	^a^ TS (MPa)	Basic Weight (g/m^2^)	Target Molecule	E_PM_ (%)	∆P (Pa)	QF (Pa^−1^)	Ref
PMIA	ESN	72.8	0.365	300–500 nm NaCl aerogel particles	99.999	92	0.183	[60]
PU	ESN	13–15	0.36	PM_1–0.5_ PM_2.5–1_	>99.00 >99.73	28	0.12	[117]
N6/PAN	ESN	-	2.94	300 nm NaCl aerosol particle	99.99	37–60	0.1163	[135]
PA-6	ESN	-	0.9	300–500 nm NaCl aerogel particles	99.996	95	>0.11	[136]
PA-56	ESN	11.02	~0.63	300–500 nm NaCl aerogel particles	99.995	111	0.108	[137]
PLA	ES	-	5.21	NaCl aerosol particles of 260 nm average diameter	99.997	165.3	0.06	[76]
PEO@PAN/PSU	ES	8.2	3.5	300–500 nm NaCl aerogel particles	99.992	95	0.1	[138]
PLLA ^b^	ES	-	-	PM_2.5_ PM_0.3_	>99.9 >99.5	≈20	-	[139]
PLA	ES	14.19	-	PM_0.3_	99.992	107	-	[131]

^a^ TS= Tensile strength, ^b^ PLLA= Poly (L-Lactic acid).

**Table 6:** Fabrication technique, physical properties and filtration performances of some modified NF membranes.

Polymer	Additive	Fabrication Technique	^a^ TS (MPa)	Target Molecule	E_PM_ (%)	∆P (Pa)	QF (Pa^−1^)	Ref.
PVDF	^b^ GPS@SiO_2_ NPs	ES	-	NaCl aerosols	99.996	14–18.5	0.14	[56]
PEI	MgTPP @EDA	ES	-	CO_2_ PM_2.5_	74	436	-	[57]
PAN	MOF	ES	-	PM	99.99	30.5	-	[140]
PAN	^c^ AgNPs ^d^ MA NPs	ES	-	PM_2.5_	99.1	-	-	[143]
PAN	MOF@ ^e^ BCP	ESN	-	Sub-100 nm particles	86.2%	51.4	0.0387	[144]
^f^ CNF	Carbon nanoparticle	ES	26.48 ± 0.4	-	99.99%	-	-	[129]
PVDF	GO	ES	3.76	PM_2.5_	99.31	28.17	0.049	[103]
^g^ PASS	GO@Ag	ES	2.39 ± 0.07	PM_0.30–2.50_	99.63 ± 0.34	79.17 ± 1.07	~0.065	[145]
PMIA	TiO_2_	ES	-	PM_2.5_	99.3	61	-	[146]
PS	HAP/ZIF-8	ES	-	PM_2.5_ PM_10_	96.68 96.98	-	-	[142]
^h^ CA, PVA, chitosan	Ag NPs	-	-	PM_2.5_	99.78	61.15	0.09	[147]

^a^ TS = tensile strength, ^b^ GPS = γ-glycidoxypropyl trimethoxysilane, ^c^ Ag NPs = silver nanoparticles, ^d^ MA NPs = magnesium aluminate nanoparticles, ^e^ BCP = bromocresol purple, ^f^ CNF = cellulose nanofiber, ^g^ PASS = poly (aryl sulfide sulfone), ^h^ CA = cellulose acetate.

**Table 7:** Filtration performance of some microporous membranes.

Polymer	Modified with/Monomer	Target Molecule	E_PM_ (%)	∆P (Pa)	QF (Pa^−1^)	Ref.
CMPs	Aminopyridine	PM_2.5_ PM_10_	≥99.57 ± 0.19 ≥99.98 ± 0.01	30–270	0.42	[149]
Tubular CMPs	Bromated monomers	PM_2.5_ PM_10_	99.97 >99	25	-	[150]
CMPs	-	PM_2.5_ PM_10_	99.7 99.9	60–550	-	[84]
CMPs	-	PM_0.3_ PM_2.5–10_	>95 >99.5	-	-	[153]
CMPs	PVP	PM_0.3_ PM_0.5_ PM_2.5–10_	95.18 98 >99	35–40	-	[155]
CMPs	Acyl functional group	PM_0.3_ PM_2.5–10_	>99.24 ± 0.13 99.99	-	-	[156]
CMP nanotube	-	PM_0.3_ PM_2.5–10_	99.4 99.9	10	-	[154]
CMP nanotube	Thiophene	PM_0.3_ PM_2.5_	99.798 ± 0.055 99.998 ± 0.002	5	2.03, 1.01	[157]

**Table 8:** Filtration performances of some hollow fiber membranes.

Polymer	Modified with	Target Molecule	E_PM_ (%)	∆P (Pa)	QF (kPa^−1^)	Ref.
PES	-	Ultrafine particles in PM_2.5_	99.995	-	-	[162]
PTFE	-	PM_0.3_ PM_2.5_	90 99.99	<400	-	[163]
PTFE	AgZrP	PM_0.3_ PM_2.5_	97.7324 99.9984	-	-	[164]
PP	-	Particle size > 60 nm 35.9–40 nm	99 82–86	-	2–28	[165]

**Table 9:** Surface contact angles and filtration performance of Janus membranes.

Polymer	Modified with	Contact Angle (Hydrophobic /Hydrophilic)	Target Molecule	E_PM_ (%)	∆P (Pa)	QF (Pa^−1^)	Ref.
PAN	β-CD/PCL/ZnO	125.6°/24.6°	PM	99.99	156.5	0.05885	[166]
	β-CD/11% PCL/2% PCL	118.1°/17.4°		99.98	165.1	0.05159	
	β-CD/mask	133.1°/41.7°		91.56	15.2	0.1626	
PAN	TiO_2_/(PVDF-HFP)@SiO_2_	150 ± 2.5°/0°	PM_0.3_	99.7	27	0.2152	[167]
PP and cotton fiber	12-hydroxystearic acid and halicin	157.1°/0°	PM_2.5_ PM_10_	93.54 98.35	57	0.072	[170]
PAN/PVP	PCL	145°/0°	Dust particles	99.98	134.7	0.065	[168]
CA	Quaternary chitosan	-	PM_0.3_ PM_1.0_ PM_2.5_	96.4 99.9 100	48	-	[169]

**Table 10:** Antibacterial activities and filtration performance of air filtration membranes.

Polymer	Modified with	Antibacterial Activity		Target Molecule	E_PM_ (%)	Ref.
		Pathogen	Antibacterial Efficiency			
PCL	^a^ HNTs-ZnO/PCL	*S. aureus*	97.9%	PM_2.5_	92.10	[183]
		*E. coli*	95.9%			
^b^ HEI-PVP	*Sophora flavescens*	*S. epidermidis*	∼99.98%	PM_0.5–20_	99.99	[189]
PU	AC/^c^ CO	*S. aureus*	11.1 (^d^ ZI)	300–500 nm NaCl	68.23	[190]
		*E. coli*	12.7 (^d^ ZI)			
PP and cotton fiber	12-hydroxystearic acid and halicin	*E. coli*	99.9999	PM_2.5_ PM_10_	93.54 98.35	[170]
CA	Quaternary chitosan	*E. coli*	98.27 ± 0.45%	PM_0.3_ PM_1.0_ PM_2.5_	96.4 99.9 100	[169]
		*S. aureus*	98.65 ± 0.26%			
PET/PVA	TiO_2_ and Ag	*E. coli*	98.7%	NaCl	99.87	[192]
		*S. aureus*	95.9%	^e^ DEHS	99.89	
Chitosan/PVA/CA	Ag NPs	*E. coli*	100–141%	PM_2.5_	99.78	[147]
		*S. aureus*				
Ethyl cellulose	Tea polyphenol	*E. coli*	99.99%	0.3 μm NaCl particles	99.991	[193]
		*S. aureus*				
PSF	Ag NPs and GO nanosheet	*S. aureus*	>99.99%	*S. aureus*	>99.9	[180]
		*E. coli*				
		*K. pneumoniae*				
		*C. albicans,*				
		*B. subtilis*				
PAN	Chitosan biguanide hydrochloride	*E. coli*	>99.99%	PM	98	[133]
		*S. aureus*				

^a^ HNTs = halloysite nanotubes, ^b^ HEI= herbal extract incorporated, ^c^ CO = cinnamon essential oil, ^d^ ZI = zone of inhibition in mm unit, ^e^ DEHS= dioctyl sebacate.

## Data Availability

No new data were created or analyzed in this study.

## References

[1] Kampa M., Castanas E. (2008). Human health effects of air pollution. Environ. Pollut..

[2] Li X., Jin L., Kan H. (2019). Air pollution: A global problem needs local fixes. Nature.

[3] Ren J., Li B., Yu D., Liu J., Ma Z. (2016). Approaches to prevent the patients with chronic airway diseases from exacerbation in the haze weather. J. Thorac. Dis..

[4] Quinn P.K., Bates T.S. (2003). North American, Asian, and Indian haze: Similar regional impacts on climate?. Geophys. Res. Lett..

[5] Britigan N., Alshawa A., Nizkorodov S.A. (2006). Quantification of ozone levels in indoor environments generated by ionization and ozonolysis air purifiers. J. Air Waste Manag. Assoc..

[6] Bhuyan C., Konwar A., Bora P., Rajguru P., Hazarika S. (2023). Cellulose nanofiber-poly (ethylene terephthalate) nanocomposite membrane from waste materials for treatment of petroleum industry wastewater. J. Hazard. Mater..

[7] Bora P., Bhuyan C., Borah A.R., Hazarika S. (2023). Carbon nanomaterials for designing next-generation membranes and their emerging applications. Chem. Commun..

[8] Goswami R., Gogoi M., Borah A., Sarmah H., Borah A.R., Feng X., Hazarika S. (2024). Quantum dot-β-Cyclodextrin nanofiller decorated thin film nanocomposite membrane for removal of cationic and anionic dyes from aqueous solution. Mater. Today Chem..

[9] Nallathambi G., Robert B., Esmeralda S.P., Kumaravel J., Parthiban V. (2020). Development of SPI/AC/PVA nano-composite for air-filtration and purification. Res. J. Text. Appar..

[10] Reena P., Gobi N., Chitralekha P., Thenmuhil D., Kamaraj V. (2021). Mesoporous titania-embedded polyacrylonitrile composite nanofibrous membrane for particulate matter filtration. J. Thermoplast. Compos. Mater..

[11] Zhu X., Feng S., Zhao S., Zhang F., Xu C., Hu M., Zhong Z., Xing W. (2020). Perfluorinated superhydrophobic and oleophobic SiO_2_@ PTFE nanofiber membrane with hierarchical nanostructures for oily fume purification. J. Membr. Sci..

[12] Yu Y., Ma Q., Zhang J.B., Liu G.B. (2020). Electrospun SiO_2_ aerogel/polyacrylonitrile composited nanofibers with enhanced adsorption performance of volatile organic compounds. Appl. Surf. Sci..

[13] Ji X., Huang J., Teng L., Li S., Li X., Cai W., Chen Z., Lai Y. (2023). Advances in particulate matter filtration: Materials, performance, and application. Green Energy Environ..

[14] Zhou Y., Liu Y., Zhang M., Feng Z., Yu D.G., Wang K. (2022). Electrospun nanofiber membranes for air filtration: A review. Nanomaterials.

[15] De Riccardis M.F. (2023). Electrospun nanofibrous membranes for air filtration: A critical review. Compounds.

[16] Valencia-Osorio L.M., Álvarez-Láinez M.L. (2021). Global view and trends in electrospun nanofiber membranes for particulate matter filtration: A review. Macromol. Mater. Eng..

[17] Lu T., Cui J., Qu Q., Wang Y., Zhang J., Xiong R., Ma W., Huang C. (2021). Multistructured electrospun nanofibers for air filtration: A review. ACS Appl. Mater. Interfaces.

[18] Deng Y., Lu T., Cui J., Samal S.K., Xiong R., Huang C. (2021). Bio-based electrospun nanofiber as building blocks for a novel eco-friendly air filtration membrane: A review. Sep. Purif. Technol..

[19] Zhang S., Ren Q., Qi H., Liu S., Liu Y. (2019). Adverse effects of fine-particle exposure on joints and their surrounding cells and microenvironment. ACS Nano.

[20] Liu H., Cao C., Huang J., Chen Z., Chen G., Lai Y. (2020). Progress on particulate matter filtration technology: Basic concepts, advanced materials, and performances. Nanoscale.

[21] Deng W., Sun Y., Yao X., Subramanian K., Ling C., Wang H., Chopra S.S., Xu B.B., Wang J.X., Chen J.F. (2022). Masks for COVID-19. Adv. Sci..

[22] Zhang R., Liu B., Yang A., Zhu Y., Liu C., Zhou G., Sun J., Hsu P.C., Zhao W., Lin D. (2018). In situ investigation on the nanoscale capture and evolution of aerosols on nanofibers. Nano Lett..

[23] Li N., Sioutas C., Cho A., Schmitz D., Misra C., Sempf J., Wang M., Oberley T., Froines J., Nel A. (2003). Ultrafine particulate pollutants induce oxidative stress and mitochondrial damage. Environ. Health Perspect..

[24] Lee C.J., Martin R.V., Henze D.K., Brauer M., Cohen A., Donkelaar A.V. (2015). Response of global particulate-matter-related mortality to changes in local precursor emissions. Environ. Sci. Technol..

[25] Li X., Zhou S., Fan W. (2016). Effect of nano-Al_2_O_3_ on the toxicity and oxidative stress of copper towards *Scenedesmus obliquus*. Int. J. Environ. Res. Public Health.

[26] Mukherjee A., Agrawal M. (2017). World air particulate matter: Sources, distribution, and health effects. Environ. Chem. Lett..

[27] Xu B., Hao J. (2017). Air quality inside subway metro indoor environment worldwide: A review. Environ. Int..

[28] Han S., Kim J., Ko S.H. (2021). Advances in air filtration technologies: Structure-based and interaction-based approaches. Mater. Today Adv..

[29] Archer B., Shaumbwa V.R., Liu D., Li M., Iimaa T., Surenjav U. (2021). Nanofibrous mats for particulate matter filtration. Ind. Eng. Chem. Res..

[30] Wang J., Zhao B., Wang S., Yang F., Xing J., Morawska L., Ding A., Kulmala M., Kerminen V.M., Kujansuu J. (2017). Particulate matter pollution over China and the effects of control policies. Sci. Total Environ..

[31] Wang C., Tu Y., Yu Z., Lu R. (2015). PM2.5 and cardiovascular diseases in the elderly: An overview. Int. J. Environ. Res. Public Health.

[32] Luo C., Zhu X., Yao C., Hou L., Zhang J., Cao J., Wang A. (2015). Short-term exposure to particulate air pollution and risk of myocardial infarction: A systematic review and meta-analysis. Environ. Sci. Pollut. Res. Int..

[33] Newby D.E., Mannucci P.M., Tell G.S., Baccarelli A.A., Brook R.D., Donaldson K., Forastiere F., Franchini M., Franco O.H., Graham I. (2015). Expert position paper on air pollution and cardiovascular disease. Eur. Heart J..

[34] Kajbafzadeh M., Brauer M., Karlen B., Carlsten C., van Eeden S., Allen R.W. (2015). The impacts of traffic-related and woodsmoke particulate matter on measures of cardiovascular health: A HEPA filter intervention study. Occup. Environ. Med..

[35] Lim S.S., Vos T., Flaxman A.D., Danaei G., Shibuya K., Adair-Rohani H., AlMazroa M.A., Amann M., Anderson H.R., Andrews K.G. (2012). A comparative risk assessment of burden of disease and injury attributable to 67 risk factors and risk factor clusters in 21 regions, 1990–2010: A systematic analysis for the Global Burden of Disease Study 2010. Lancet.

[36] Apte J.S., Marshall J.D., Cohen A.J., Brauer M. (2015). Addressing global mortality from ambient PM2.5. Environ. Sci. Technol..

[37] Maestas M.M., Brook R.D., Ziemba R.A., Li F., Crane R.C., Klaver Z.M., Bard R.L., Spino C.A., Adar S.D., Morishita M. (2019). Reduction of personal PM2.5 exposure via indoor air filtration systems in Detroit: An intervention study. J. Expo. Sci. Environ. Epidemiol..

[38] Zhou M., Wang H., Zeng X., Yin P., Zhu J., Chen W., Li X., Wang L., Wang L., Liu Y. (2019). Mortality, morbidity, and risk factors in China and its provinces, 1990–2017: A systematic analysis for the Global Burden of Disease Study 2017. Lancet.

[39] Verdoni L., Mazza A., Gervasoni A., Martelli L., Ruggeri M., Ciuffreda M., Bonanomi E., D’Antiga L. (2020). An outbreak of severe Kawasaki-like disease at the Italian epicentre of the SARS-CoV-2 epidemic: An observational cohort study. Lancet.

[40] Corman V.M., Landt O., Kaiser M., Molenkamp R., Meijer A., Chu D.K., Bleicker T., Brünink S., Schneider J., Schmidt M.L. (2020). Detection of 2019 novel coronavirus (2019-nCoV) by real-time RT-PCR. Eurosurveillance.

[41] Setti L., Passarini F., De Gennaro G., Barbieri P., Perrone M.G., Borelli M., Palmisani J., Di Gilio A., Torboli V., Fontana F. (2020). SARS-CoV-2RNA found on particulate matter of Bergamo in Northern Italy: First evidence. Environ. Res..

[42] Liu C., Hsu P.C., Lee H.W., Ye M., Zheng G., Liu N., Li W., Cui Y. (2015). Transparent air filter for high-efficiency PM_2.5_ capture. Nat. Commun..

[43] Grantz D.A., Garner J.H.B., Johnson D.W. (2003). Ecological effects of particulate matter. Environ. Int..

[44] Thangavel P., Park D., Lee Y.C. (2022). Recent insights into particulate matter (PM_2.5_)-mediated toxicity in humans: An overview. Int. J. Environ. Res. Public Health.

[45] Vijayan V.K., Paramesh H., Salvi S.S., Dalal A.A.K. (2015). Enhancing indoor air quality—The air filter advantage. Lung India.

[46] Roy A., Mishra C., Jain S., Solanki N. (2019). A review of general and modern methods of air purification. J. Therm. Eng..

[47] Chambre A. (2014). Effects of Carbon Filtration Type on Filter Efficiency and Efficacy: Granular Loose-Fill vs. Bonded Filters.

[48] Zhao Y., Aarnink A.J., Hofschreuder P., Koerkamp P.W.G. (2009). Evaluation of an impaction and a cyclone pre-separator for sampling high PM10 and PM2. 5 concentrations in livestock houses. J. Aerosol Sci..

[49] Robert B., Nallathambi G. (2020). A concise review on electrospun nanofibres/nanonets for filtration of gaseous and solid constituents (PM2.5) from polluted air. Colloid Interface Sci. Commun..

[50] Li P., Wang C., Zhang Y., Wei F. (2014). Air filtration in the free molecular flow regime: A review of high-efficiency particulate air filters based on carbon nanotubes. Small.

[51] Hutten I. (2007). Fabrics. Handbook of Nonwoven Filter Media.

[52] Chen C.Y. (1955). Filtration of aerosols by fibrous media. Chem. Rev..

[53] Bui T.T., Shin M.K., Jee S.Y., Long D.X., Hong J., Kim M.G. (2022). Ferroelectric PVDF nanofiber membrane for high-efficiency PM0.3 air filtrations with low air flow resistance. Colloids Surf. A Physicochem. Eng. Asp..

[54] Zhao K., Huang J., Mao J., Bao Z., Chen Z., Lai Y. (2020). Charged graphene aerogel filter enabled superior particulate matter removal efficiency in harsh environment. Chem. Eng. J..

[55] Xie Y., Guo F., Mao J., Huang J., Chen Z., Jiang Y., Lai Y. (2021). Freestanding MoS_2_@ carbonized cellulose aerogel derived from waste cotton for sustainable and highly efficient particulate matter capturing. Sep. Purif. Technol..

[56] Ding X., Li Y., Si Y., Yin X., Yu J., Ding B. (2019). Electrospun polyvinylidene fluoride/SiO_2_ nanofibrous membranes with enhanced electret property for efficient air filtration. Compos. Commun..

[57] Ramachandran S., Rajiv S. (2020). Ethylenediamine functionalized metalloporphyrin loaded nanofibrous membrane: A new strategic approach to air filtration. J. Inorg. Organomet. Polym. Mater..

[58] Nakashima H., Ooshima T. (2007). Analysis of inorganic antimicrobial agents in antimicrobial products: Evaluation of a screening method by X-ray fluorescence spectrometry and the measurement of metals by inductively coupled plasma atomic emission spectroscopy. J. Health Sci..

[59] Martínez-Camacho A.P., Cortez-Rocha M.O., Castillo-Ortega M.M., Burgos-Hernández A., Ezquerra-Brauer J.M., Plascencia-Jatomea M. (2011). Antimicrobial activity of chitosan nanofibers obtained by electrospinning. Polym. Int..

[60] Zhang S., Liu H., Yin X., Li Z., Yu J., Ding B. (2017). Tailoring mechanically robust poly (m-phenylene isophthalamide) nanofiber/nets for ultrathin high-efficiency air filter. Sci. Rep..

[61] Leung W.W.F., Hung C.H., Yuen P.T. (2010). Effect of face velocity, nanofiber packing density and thickness on filtration performance of filters with nanofibers coated on a substrate. Sep. Purif. Technol..

[62] Xia T., Chen C. (2021). Evolution of pressure drop across electrospun nanofiber filters clogged by solid particles and its influence on indoor particulate air pollution control. J. Hazard. Mater..

[63] Han N., Lee Y.S., Kaang B.K., Jang W., Koo H.Y., Choi W.S. (2019). A lottery draw machine-inspired movable air filter with high removal efficiency and low pressure drop at a high flow rate. J. Mater. Chem. A.

[64] Cao J., Cheng Z., Kang L., Lin M., Han L. (2020). Patterned nanofiber air filters with high optical transparency, robust mechanical strength, and effective PM2.5 capture capability. RSC Adv..

[65] Zhang L., Yuan W.L., Zhang Z., Zhang G.H., Chen H., Zhao N., He L., Tao G.H. (2019). Self-assembled ionic nanofibers derived from amino acids for high-performance particulate matter removal. J. Mater. Chem. A.

[66] Hao Z., Wu J., Wang C., Liu J. (2019). Electrospun polyimide/metal–organic framework nanofibrous membrane with superior thermal stability for efficient PM2.5 capture. ACS Appl. Mater. Interfaces.

[67] Li C.X., Kuang S.Y., Chen Y.H., Wang Z.L., Li C., Zhu G. (2018). In situ active poling of nanofiber networks for gigantically enhanced particulate filtration. ACS Appl. Mater. Interfaces.

[68] Wang N., Raza A., Si Y., Yu J., Sun G., Ding B. (2013). Tortuously structured polyvinyl chloride/polyurethane fibrous membranes for high-efficiency fine particulate filtration. J. Colloid Interface Sci..

[69] Zhang Q., Li Q., Young T.M., Harper D.P., Wang S. (2019). A novel method for fabricating an electrospun poly(vinyl alcohol)/cellulose nanocrystals composite nanofibrous filter with low air resistance for high-efficiency filtration of particulate matter. ACS Sustain. Chem. Eng..

[70] Rajak A., Hapidin D.A., Iskandar F., Munir M.M., Khairurrijal K. (2020). Electrospun nanofiber from various sources of expanded polystyrene (EPS) waste and their characterization as potential air filter media. Waste Manag..

[71] Lou Y., Ding S., Wang B., Wang J., Sun Q., Jin X., Li X. (2021). Controllable morphology of electrospun nanofiber membranes with tunable groove structure and the enhanced filtration performance for ultrafine particulates. Nanotechnology.

[72] Zuo F., Zhang S., Liu H., Fong H., Yin X., Yu J., Ding B. (2017). Free-standing polyurethane nanofiber/nets air filters for effective PM capture. Small.

[73] Balgis R., Murata H., Goi Y., Ogi T., Okuyama K., Bao L. (2017). Synthesis of dual-size cellulose–polyvinylpyrrolidone nanofiber composites via one-step electrospinning method for high-performance air filter. Langmuir.

[74] Fan Q., Liang W., Fan T.T., Li X., Yan S.Y., Yu M., Ning X., Long Y.Z. (2020). Polyvinylidene fluoride composite nanofibrous filter for high-efficiency PM_2.5_ capture. Compos. Commun..

[75] Guo J., Hanif A., Shang J., Deka B.J., Zhi N., An A.K. (2021). PAA@ZIF-8 incorporated nanofibrous membrane for high-efficiency PM2.5 capture. Chem. Eng. J..

[76] Wang Z., Zhao C., Pan Z. (2015). Porous bead-on-string poly(lactic acid) fibrous membranes for air filtration. J. Colloid Interface Sci..

[77] Jackiewicz-Zagórska A., Mika K., Penconek A., Moskal A. (2022). Non-woven filters made of PLA via solution blowing process for effective aerosol nanoparticle filtration. Processes.

[78] Xie X., Zheng Z., Wang X., Kaplan D.L. (2021). Low-density silk nanofibrous aerogels: Fabrication and applications in air filtration and oil/water purification. ACS Nano.

[79] Choi S., Jeon H., Jang M., Kim H., Shin G., Koo J.M., Lee M., Sung H.K., Eom Y., Yang H.S. (2021). Biodegradable, efficient, and breathable multi-use face mask filter. Adv. Sci..

[80] Xiong Z., Lin J., Li X., Bian F., Wang J. (2021). Hierarchically structured nanocellulose-implanted air filters for high-efficiency particulate matter removal. ACS Appl. Mater. Interfaces.

[81] Yu X., Li C., Tian H., Yuan L., Xiang A., Li J., Wang C., Rajulu A.V. (2020). Hydrophobic cross-linked zein-based nanofibers with efficient air filtration and improved moisture stability. Chem. Eng. J..

[82] Tian H., Fu X., Zheng M., Wang Y., Li Y., Xiang A., Zhong W.H. (2018). Natural polypeptides treat pollution complex: Moisture-resistant multifunctional protein nanofabrics for sustainable air filtration. Nano Res..

[83] Senthil R., Sumathi V., Tamilselvi A., Kavukcu S.B., Aruni A.W. (2022). Functionalized electrospun nanofibers for high efficiency removal of particulate matter. Sci. Rep..

[84] Wang Y., Su Y., Yang L., Su M., Niu Y., Liu Y., Sun H., Zhu Z., Liang W., Li A. (2022). Highly efficient removal of PM and VOCs from air by a self-supporting bifunctional conjugated microporous polymers membrane. J. Membr. Sci..

[85] Wang J.T., Ge Y.Y., He Y., Xu M.X., Cui X.M. (2019). A porous gradient geopolymer-based tube membrane with high PM removal rate for air pollution. J. Clean. Prod..

[86] Deng Q., Li J., Li X., Du X., Wu L., Wang J., Wang X. (2024). Incorporating nano-ZnCo-ZIF particles in electrospinning polylactide membranes to improve their filtration and antibacterial performances. Polym. Bull..

[87] Yang L., Niu C., Cao X., Zhu Z., Sun H., Liang W., Li J., Li A. (2022). Efficient capture of airborne PM by membranes based on holey reduced graphene oxide nanosheets. J. Environ. Chem. Eng..

[88] Koo W.T., Jang J.S., Qiao S., Hwang W., Jha G., Penner R.M., Kim I.D. (2018). Hierarchical metal–organic framework-assembled membrane filter for efficient removal of particulate matter. ACS Appl. Mater. Interfaces.

[89] Karmakar K., Hossain M., Sarkar P., Rao K.D.M. (2023). Silk nanofibrous network for biodegradable PM0.3 filters rejuvenated with in-situ Joule heating for self-sanitization. SSRN Preprint.

[90] Kaang B.K., Lee H.B., Koo H.Y., Choi W.S. (2020). Wastepaper-Based Cylindrical Hollow Air Filter Module for the Removal of Particulate Matter (PM_10_ and PM_2.5_) and HCHO. ACS Sustain. Chem. Eng..

[91] Woo H., Yoo D.K., Jhung S.H. (2020). Highly improved performance of cotton air filters in particulate matter removal by the incorporation of metal–organic frameworks with functional groups capable of large charge separation. ACS Appl. Mater. Interfaces.

[92] Ahn J., Park H., Ryu T., Park J. (2023). Core–shell structured mixed cellulose ester–alumina composite membranes for air filters with improved environmental resistance. Sep. Purif. Technol..

[93] Hao D., Yu B., Zhao T., Chen M. (2025). Filtration performance of NH_2_-MIL-53/chitosan-modified paper-based functional materials against airborne particulate matter. ACS Omega.

[94] Li S., Xie D., Song L., Yang C., Yuan Z. (2025). Rapid and sustainable fabrication of antibacterial chitosan/PVA–SiO_2_ nanofiber air filters by needleless electrospinning. AIP Adv..

[95] Tang X., Li N., Pang H. (2022). Metal–organic frameworks-derived metal phosphides for electrochemistry application. Green Energy Environ..

[96] Ma S., Zhang M., Nie J., Tan J., Yang B., Song S. (2019). Design of double-component metal–organic framework air filters with PM_2.5_ capture, gas adsorption and antibacterial capacities. Carbohydr. Polym..

[97] Wang H., Xu H., Li H., Liu X., Du Z., Yu W. (2020). Electrospun polyurethane/zeolitic imidazolate framework nanofibrous membrane with superior stability for filtering performance. ACS Appl. Polym. Mater..

[98] Woo H.C., Yoo D.K., Jhung S.H. (2021). Particulate matters removal by using cotton coated with isomeric metal–organic frameworks (MOFs): Effect of voidage of MOFs on removal. J. Ind. Eng. Chem..

[99] Yoo D.K., Woo H.C., Jhung S.H. (2021). Removal of particulate matters by using zeolitic imidazolate framework-8s (ZIF-8s) coated onto cotton: Effect of the pore size of ZIF-8s on removal. ACS Appl. Mater. Interfaces.

[100] Mao J., Ge M., Chen I.W.P., Ng Y.H., Zhu T., Liu H., Huang J., Cai W., Lai Y. (2021). In situ recycling of particulate matter for a high-performance supercapacitor and oxygen evolution reaction. Mater. Chem. Front..

[101] Wang S., Bai J., Innocent M.T., Wang Q., Xiang H., Tang J., Zhu M. (2022). Lignin-based carbon fibers: Formation, modification and potential applications. Green Energy Environ..

[102] Yuan K., Feng S., Zhang F., Zhong Z., Xing W. (2020). Steric configuration-controllable carbon nanotubes-integrated SiC membrane for ultrafine particles filtration. Ind. Eng. Chem. Res..

[103] Chen M., Jiang J., Feng S., Low Z.X., Zhong Z., Xing W. (2021). Graphene oxide functionalized polyvinylidene fluoride nanofibrous membranes for efficient particulate matter removal. J. Membr. Sci..

[104] Wen T.Y., Wang H.C., Krichtafovitch I., Mamishev A.V. (2015). Novel electrodes of an electrostatic precipitator for air filtration. J. Electrost..

[105] Jeong S., Cho H., Han S., Won P., Lee H., Hong S., Yeo J., Kwon J., Ko S.H. (2017). High efficiency, transparent, reusable, and active PM_2.5_ filters by hierarchical Ag nanowire percolation network. Nano Lett..

[106] Huang W.R., He Z., Wang J.L., Liu J.W., Yu S.H. (2019). Mass production of nanowire–nylon flexible transparent smart windows for PM_2.5_ capture. iScience.

[107] Bai Y., Han C.B., He C., Gu G.Q., Nie J.H., Shao J.J., Xiao T.X., Deng C.R., Wang Z.L. (2018). Washable multilayer triboelectric air filter for efficient particulate matter PM_2.5_ removal. Adv. Funct. Mater..

[108] Han S., Hong S., Ham J., Yeo J., Lee J., Kang B., Lee P., Kwon J., Lee S.S., Yang M.Y. (2014). Fast plasmonic laser nanowelding for a Cu-nanowire percolation network for flexible transparent conductors and stretchable electronics. Adv. Mater..

[109] Xiong Z.C., Yang R.L., Zhu Y.J., Chen F.F., Dong L.Y. (2017). Flexible hydroxyapatite ultralong nanowire-based paper for highly efficient and multifunctional air filtration. J. Mater. Chem. A.

[110] Souzandeh H., Wang Y., Netravali A.N., Zhong W.H. (2019). Towards sustainable and multifunctional air-filters: A review on biopolymer-based filtration materials. Polym. Rev..

[111] Jin Y., Liu H., Feng M., Ma Q., Wang B. (2024). Metal-organic frameworks for air pollution purification and detection. Adv. Funct. Mater..

[112] Attia Y.A., Ezet A.E., Saeed S., Galmed A.H. (2024). Nano carbon-modified air purification filters for removal and detection of particulate matters from ambient air. Sci. Rep..

[113] Peng Z., Liu X., Zhang W., Zeng Z., Liu Z., Zhang C., Liu Y., Shao B., Liang Q., Tang W. (2020). Advances in the application, toxicity and degradation of carbon nanomaterials in environment: A review. Environ. Int..

[114] Kim J., Park J., Tsai P.J., Yoon C. (2024). The effects of temperature and humidity on electrostatic changes in respirators and their filtration efficiency. Indoor Air.

[115] Ye S., Rathmell A.R., Chen Z., Stewart I.E., Wiley B.J. (2014). Metal nanowire networks: The next generation of transparent conductors. Adv. Mater..

[116] Thavasi V., Singh G., Ramakrishna S. (2008). Electrospun nanofibers in energy and environmental applications. Energy Environ. Sci..

[117] Zhu M., Han J., Wang F., Shao W., Xiong R., Zhang Q., Pan H., Yang Y., Samal S.K., Zhang F. (2017). Electrospun nanofibers membranes for effective air filtration. Macromol. Mater. Eng..

[118] Nakajima T., Kajiwara K., McIntyre J.E. (1994). Advanced Fiber Spinning Technology.

[119] Borah A.R., Hazarika P., Duarah R., Goswami R., Hazarika S. (2024). Biodegradable electrospun membranes for sustainable industrial applications. ACS Omega.

[120] Uppal R., Bhat G., Eash C., Akato K. (2013). Meltblown nanofiber media for enhanced quality factor. Fibers Polym..

[121] Huan S., Liu G., Han G., Cheng W., Fu Z., Wu Q., Wang Q. (2015). Effect of experimental parameters on morphological, mechanical and hydrophobic properties of electrospun polystyrene fibers. Materials.

[122] Wang N., Si Y., Wang N., Sun G., El-Newehy M., Al-Deyab S.S., Ding B. (2014). Multilevel structured polyacrylonitrile/silica nanofibrous membranes for high-performance air filtration. Sep. Purif. Technol..

[123] Yoon K., Kim K., Wang X., Fang D., Hsiao B.S., Chu B. (2006). High flux ultrafiltration membranes based on electrospun nanofibrous PAN scaffolds and chitosan coating. Polymer.

[124] Wang N., Cai M., Yang X., Yang Y. (2018). Electret nanofibrous membrane with enhanced filtration performance and wearing comfortability for face mask. J. Colloid Interface Sci..

[125] Liu Y., Park M., Ding B., Kim J., El-Newehy M., Al-Deyab S.S., Kim H.Y. (2015). Facile electrospun polyacrylonitrile/poly(acrylic acid) nanofibrous membranes for high efficiency particulate air filtration. Fibers Polym..

[126] Robert B., Nallathambi G. (2023). Structural design and development of multilayered polymeric nanofibrous membrane for multifaceted air filtration/purification applications. Polym.-Plast. Technol. Mater..

[127] Oh H.J., Pant H.R., Kang Y.S., Jeon K.S., Pant B., Kim C.S., Kim H.Y. (2012). Synthesis and characterization of spider-web-like electrospun mats of meta-aramid. Polym. Int..

[128] Yao L., Lee C., Kim J. (2010). Fabrication of electrospun meta-aramid nanofibers in different solvent systems. Fibers Polym..

[129] Senthil R., Vedakumari W.S., Kavukcu S.B. (2024). Wood-based cellulose nanofiber membrane: A novel approach to high-performance air filters. Cellulose.

[130] Khan M.J., Karim Z., Pongchaikul P., Posoknistakul P., Intra P., Laosiripojana N., Wu K.C.W., Sakdaronnarong C. (2024). Nitrogen and sulfur doped carbon dots coupled cellulose nanofibers: A surface functionalized nanocellulose membrane for air filtration. J. Taiwan Inst. Chem. Eng..

[131] Shao W., Niu J., Han R., Liu S., Wang K., Cao Y., Han P., Li X., Zhang H., Yu H. (2023). Electrospun multiscale poly(lactic acid) nanofiber membranes with a synergistic antibacterial effect for air-filtration applications. ACS Appl. Polym. Mater..

[132] Zhao Y., Ming J., Cai S., Wang X., Ning X. (2024). One-step fabrication of polylactic acid (PLA) nanofibrous membranes with spider-web-like structure for high-efficiency PM_0.3_ capture. J. Hazard. Mater..

[133] Wang G., Xiao D., Fang Y., Ning G., Ye J. (2024). Polarity-dominated chitosan biguanide hydrochloride-based nanofibrous membrane with antibacterial activity for long-lasting air filtration. Int. J. Biol. Macromol..

[134] Shen R., Shao Z., Chen R., Wang Q., Gui Z., Qi Y., Song W., Liu Y., Zheng G. (2024). Fully bio-based zein/chitosan hydrochloride/phloretin bimodal fibrous membrane for high-performance and antibacterial air filtration based on green electrospinning. Sep. Purif. Technol..

[135] Wang N., Yang Y., Al-Deyab S.S., El-Newehy M., Yu J., Ding B. (2015). Ultra-light 3D nanofibre-nets binary structured nylon 6–polyacrylonitrile membranes for efficient filtration of fine particulate matter. J. Mater. Chem. A.

[136] Zhang S., Liu H., Zuo F., Yin X., Yu J., Ding B. (2017). A controlled design of ripple-like polyamide-6 nanofiber/nets membrane for high-efficiency air filter. Small.

[137] Liu B., Zhang S., Wang X., Yu J., Ding B. (2015). Efficient and reusable polyamide-56 nanofiber/nets membrane with bimodal structures for air filtration. J. Colloid Interface Sci..

[138] Zhang S., Liu H., Yin X., Yu J., Ding B. (2016). Anti-deformed polyacrylonitrile/polysulfone composite membrane with binary structures for effective air filtration. ACS Appl. Mater. Interfaces.

[139] Li L., Gao Y., Nie G., Yan X., Wang S., Zhang T., Ramakrishna S., Long Y.Z., Han W. (2024). Biodegradable poly(L-lactic acid) fibrous membrane with ribbon-structured fibers and ultrafine nanofibers enhances air filtration performance. Small.

[140] Wang X., Xu W., Yan X., Chen Y., Guo M., Zhou G., Tong S., Ge M., Liu Y., Chen C. (2019). MOF-based fibrous membranes adsorb PM efficiently and capture toxic gases selectively. Nanoscale.

[141] Kim D.I., Park J.H., Kim S.D., Lee J.Y., Yim J.H., Jeon J.K., Park S.H., Park Y.K. (2011). Comparison of removal ability of indoor formaldehyde over different materials functionalized with various amine groups. J. Ind. Eng. Chem..

[142] Jung S., Yusuf M., Son Y., Han S., Lee H., Mahadadalkar M.A., Park S., Youn B., Lee J.M., Park K.H. (2024). An efficient atmospheric pollution control using hierarchical porous nanofibers containing zeolitic-imidazolate-frameworks and hydroxyapatite nanoparticles. J. Environ. Chem. Eng..

[143] Selvam A.K., Baskar D., Nallathambi G. (2021). Layer by layer nanocomposite filter for ABC filtration. Chem. Eng. Commun..

[144] Lee J.H., Oh H.J., Park Y.K., Kim Y., Lee G., Doh S.J., Lee W., Choi S.J., Yoon K.R. (2023). Multi-scale nanofiber membrane functionalized with metal-organic frameworks for efficient filtration of both PM_2.5_ and CH_3_CHO with colorimetric NH_3_ detection. Chem. Eng. J..

[145] Su Q., Huang Y., Wei Z., Zhu C., Zeng W., Wang S., Long S., Zhang G., Yang J., Wang X. (2023). A novel multi-gradient PASS nanofibrous membrane with outstanding particulate matter removal efficiency and excellent antimicrobial property. Sep. Purif. Technol..

[146] Wen Y., Hu Q., Wang X., Zhang W., Chen M. (2024). Electrospun poly(m-phenyleneisophthalamide)/TiO_2_ nanofiber membranes for particulate matter removal under high-temperature conditions. ACS Appl. Polym. Mater..

[147] Chueachot R., Promarak V., Saengsuwan S. (2024). Enhancing antibacterial activity and air filtration performance in electrospun hybrid air filters of chitosan (CS)/AgNPs/PVA/cellulose acetate: Effect of CS/AgNPs ratio. Sep. Purif. Technol..

[148] Jiang J.X., Su F., Niu H., Wood C.D., Campbell N.L., Khimyak Y.Z., Cooper A.I. (2008). Conjugated microporous poly(phenylene butadiynylene)s. Chem. Commun..

[149] Lei Y., Tian Z., Sun H., Zhu Z., Liang W., Li A. (2021). Self-cleaning and flexible filters based on aminopyridine conjugated microporous polymer nanotubes for bacteria sterilization and efficient PM_2.5_ capture. Sci. Total Environ..

[150] Tian Z., Lei Y., Fan Y., Zhou P., Liu F., Zhu Z., Sun H., Liang W., Li A. (2021). Efficient capture of PM_2.5_ by intertwined tubular conjugated microporous polymer-based filters with high stability in a humid environment. J. Mater. Chem. A.

[151] Jiang J.X., Su F., Trewin A., Wood C.D., Campbell N.L., Niu H., Dickinson C., Ganin A.Y., Rosseinsky M.J., Khimyak Y.Z. (2007). Conjugated microporous poly(aryleneethynylene) networks. Angew. Chem..

[152] Fan W.J., Kang Z., Zhu W.Q., Ding Y.N., Xu H.Y., Tan D.Z., Chen Y.G. (2019). Conjugated microporous polymers as novel adsorbent materials for VOCs capture: A computational study. Comput. Mater. Sci..

[153] Wang Y., Yang L., Cao X., Chan W., Jing Y., Sun H., Zhu Z., Liang W., Li J., Li A. (2023). Removal of PM and oil mist from automobile exhaust by a “hamburger”-structured conjugated microporous polymer membrane. Eur. Polym. J..

[154] Tian Z., Lei Y., Ye X., Fan Y., Zhou P., Zhu Z., Sun H., Liang W., Li A. (2022). Efficient capture of airborne PM by nanotubular conjugated microporous polymer-based filters under harsh conditions. J. Hazard. Mater..

[155] Yang L., Niu C., Cao X., Wang Y., Zhu Z., Sun H., Liang W., Li J., Li A. (2023). Mechanically robust conjugated microporous polymer membranes prepared using polyvinylpyrrolidone (PVP) electrospun nanofibers as a template for efficient PM capture. J. Colloid Interface Sci..

[156] Chai B., Wang S., Li Z., Jiang Y., Liu X., Cui M., Yu X., Xu Y., Lei Y., Zhao L. (2024). Simple synthesis of acyl coupling conjugated microporous polymer monolithic material for synergistic capture of PM and CO_2_ in flue gas and process simulation. Fuel.

[157] Lei Y., Tian Z., Sun H., Liu F., Zhu Z., Liang W., Li A. (2021). Low-resistance thiophene-based conjugated microporous polymer nanotube filters for efficient particulate matter capture and oil/water separation. ACS Appl. Mater. Interfaces.

[158] Peng N., Widjojo N., Sukitpaneenit P., Teoh M.M., Lipscomb G.G., Chung T.S., Lai J.Y. (2012). Evolution of polymeric hollow fibers as sustainable technologies: Past, present, and future. Prog. Polym. Sci..

[159] Liu Y., Feng X., Lawless D. (2006). Separation of gasoline vapor from nitrogen by hollow fiber composite membranes for VOC emission control. J. Membr. Sci..

[160] Gogoi M., Goswami R., Borah A., Bhuyan C., Sarmah H., Hazarika S. (2022). Functionalized multi-walled carbon nanotube thin-layered hollow fiber membrane for enantioselective permeation of racemic β-substituted-α-amino acids. J. Chem. Sci..

[161] Li X., Cai T., Chung T.S. (2014). Anti-fouling behavior of hyperbranched polyglycerol-grafted poly(ether sulfone) hollow fiber membranes for osmotic power generation. Environ. Sci. Technol..

[162] Li M., Feng Y., Wang K., Yong W.F., Yu L., Chung T.S. (2017). Novel hollow fiber air filters for the removal of ultrafine particles in PM_2.5_ with repetitive usage capability. Environ. Sci. Technol..

[163] Xu H., Jin W., Wang F., Li C., Wang J., Zhu H., Guo Y. (2018). Preparation and properties of PTFE hollow fiber membranes for the removal of ultrafine particles in PM_2.5_ with repetitive usage capability. RSC Adv..

[164] Xu H., Chen X., Chen M., Luo J., Jin W., Zhu H., Guo Y. (2022). Development of antibacterial PTFE hollow fiber membranes containing silver-carried zirconium phosphate as air filtration units for the removal of ultrafine particles. Fibers Polym..

[165] Bulejko P., Dohnal M., Pospíšil J., Svěrák T. (2018). Air filtration performance of symmetric polypropylene hollow-fiber membranes for nanoparticle removal. Sep. Purif. Technol..

[166] Xu W., Chen Y., Liu Y. (2021). Directional water transfer Janus nanofibrous porous membranes for particulate matter filtration and volatile organic compound adsorption. ACS Appl. Mater. Interfaces.

[167] Cui W., Fan T., Li Y., Wang X., Liu X., Lu C., Ramakrishna S., Long Y.Z. (2022). Robust functional Janus nanofibrous membranes for efficient harsh environmental air filtration and oil/water separation. J. Membr. Sci..

[168] Zhou G., Jiang L., Qu X., Sun Y., Zhu J., Li X., Ma C., Liu R., Ramakrishna S. (2025). A porous Janus nanofiber membrane with unidirectional water vapor transport for efficient dust personal protection. Sep. Purif. Technol..

[169] Yan G., Yang Z., Zhang X., Li H., Wang L., Li Z., Chen J., Wu Y. (2023). Antibacterial biodegradable nanofibrous membranes by hybrid needleless electrospinning for high-efficiency particulate matter removal. Chem. Eng. J..

[170] Deng T., Chen Y., Liu Y., Shang Z., Gong J. (2022). Constructing Janus Microsphere Membranes for Particulate Matter Filtration, Directional Water Vapor Transfer, and High-Efficiency Broad-Spectrum Sterilization. Small.

[171] Zhang P., Zhang S., Wan D., Zhang P., Zhang Z., Shao G. (2020). Multilevel polarization-fields enhanced capture and photocatalytic conversion of particulate matter over flexible Schottky-junction nanofiber membranes. J. Hazard. Mater..

[172] Ji X., Yang Y., Gou Y., Yang Y., Li W., Huang J., Cai W., Lai Y. (2023). Electrospun heterojunction nanofibrous membranes for photoinduced enhancement of fine particulate matter capture in harsh environment. Sep. Purif. Technol..

[173] Shi S., Si Y., Han Y., Wu T., Iqbal M.I., Fei B., Li R.K., Hu J., Qu J. (2022). Recent progress in protective membranes fabricated via electrospinning: Advanced materials, biomimetic structures, and functional applications. Adv. Mater..

[174] Han Y., Xu Y., Zhang S., Li T., Ramakrishna S., Liu Y. (2020). Progress of improving mechanical strength of electrospun nanofibrous membranes. Macromol. Mater. Eng..

[175] Shiohara A., Prieto-Simon B., Voelcker N.H. (2021). Porous polymeric membranes: Fabrication techniques and biomedical applications. J. Mater. Chem. B.

[176] Lau H.S., Yong W.F. (2021). Recent progress and prospects of polymeric hollow fiber membranes for gas application, water vapor separation and particulate matter removal. J. Mater. Chem. A.

[177] Yang H.C., Xie Y., Hou J., Cheetham A.K., Chen V., Darling S.B. (2018). Janus membranes: Creating asymmetry for energy efficiency. Adv. Mater..

[178] Liu R., Ji D., Zhou G., Liu Z., Xu Q., Ramakrishna S. (2021). Electrospun nanofibers for personal protection in mines. Chem. Eng. J..

[179] Li Y., Yin X., Yu J., Ding B. (2019). Electrospun nanofibers for high-performance air filtration. Compos. Commun..

[180] Liu H., Lai W., Shi Y., Tian L., Li K., Bian L., Xi Z., Lin B. (2024). Ag-decorated electrospun polymer/GO fibrous membranes for simultaneous bacterial filtration and termination. J. Membr. Sci..

[181] Tekin D., Birhan D., Kiziltas H. (2020). Thermal, photocatalytic, and antibacterial properties of calcinated nano-TiO_2_/polymer composites. Mater. Chem. Phys..

[182] Nabila M.I., Kannabiran K. (2018). Biosynthesis, characterization and antibacterial activity of copper oxide nanoparticles (CuO NPs) from actinomycetes. Biocatal. Agric. Biotechnol..

[183] Tian G., Huang Z., Wang H., Cui C., Zhang Y. (2022). Polycaprolactone nanofiber membrane modified with halloysite and ZnO for anti-bacterial and air filtration. Appl. Clay Sci..

[184] Zhang S., Yang X., Tang B., Yuan L., Wang K., Liu X., Zhu X., Li J., Ge Z., Chen S. (2018). New insights into synergistic antimicrobial and antifouling cotton fabrics via dually finished with quaternary ammonium salt and zwitterionic sulfobetaine. Chem. Eng. J..

[185] Tian C., Wu F., Jiao W., Liu X., Yin X., Si Y., Yu J., Ding B. (2021). Antibacterial and antiviral N-halamine nanofibrous membranes with nanonet structure for bioprotective applications. Compos. Commun..

[186] Saikaew R., Intasanta V. (2021). Versatile nanofibrous filters against fine particulates and bioaerosols containing tuberculosis and virus: Multifunctions and scalable processing. Sep. Purif. Technol..

[187] Qin X., Xiong Y., Xuan S., Kang J., Wang D., Wang H., Wang L., Wu Z. (2024). Nanocellulose-reinforced air filter with gradient hierarchical structure for highly effective and reusable antibacterial air filtration. J. Membr. Sci..

[188] Khan M., Khan M.S.A., Borah K.K., Goswami Y., Hakeem K.R., Chakrabartty I. (2021). The potential exposure and hazards of metal-based nanoparticles on plants and environment, with special emphasis on ZnO NPs, TiO_2_ NPs, and AgNPs: A review. Environ. Adv..

[189] Choi J., Yang B.J., Bae G.N., Jung J.H. (2015). Herbal extract incorporated nanofiber fabricated by an electrospinning technique and its application to antimicrobial air filtration. ACS Appl. Mater. Interfaces.

[190] Son B.C., Park C.H., Kim C.S. (2020). Fabrication of antimicrobial nanofiber air filter using activated carbon and cinnamon essential oil. J. Nanosci. Nanotechnol..

[191] Zhang X., Dai K., Liu C., Hu H., Luo F., Qi Q., Wang L., Ye F., Jin J., Tang J. (2021). Berberine-coated biomimetic composite microspheres for simultaneously hemostatic and antibacterial performance. Polymers.

[192] Zhou G., Xu Z., Zhang Y., Liu J., Jiang L., Liu R., Wang Y. (2024). Effect of different antibacterial agents doping in PET-based electrospun nanofibrous membranes on air filtration and antibacterial performance. Environ. Res..

[193] Shao Z., Xie J., Jiang J., Shen R., Gui Z., Li H., Wang X., Li W., Guo S., Liu Y. (2024). Research on topological effect of natural small molecule and high–performance antibacterial air filtration application by electrospinning. Sci. Total Environ..

[194] Chen F., Ji Z., Qi Q. (2019). Effect of surface wettability on filtration performance of gas–liquid coalescing filters. Powder Technol..

[195] Li P., Zong Y., Zhang Y., Yang M., Zhang R., Li S., Wei F. (2013). In situ fabrication of depth-type hierarchical CNT/quartz fiber filters for high-efficiency filtration of sub-micron aerosols and high water repellency. Nanoscale.

[196] Jiang P., Zhao X., Li Y., Liao Y., Hua T., Yin X., Yu J., Ding B. (2017). Moisture and oily molecules stable nanofibrous electret membranes for effectively capturing PM_2.5_. Compos. Commun..

[197] Zhao X., Li Y., Hua T., Jiang P., Yin X., Yu J., Ding B. (2017). Cleanable air filter transferring moisture and effectively capturing PM_2.5_. Small.

[198] Wei X., Chen F., Wang H., Zhou H., Ji Z., Lin T. (2018). Efficient removal of aerosol oil-mists using superoleophobic filters. J. Mater. Chem. A.

[199] Zhang R., Liu C., Hsu P.C., Zhang C., Liu N., Zhang J., Lee H.R., Lu Y., Qiu Y., Chu S. (2016). Nanofiber air filters with high-temperature stability for efficient PM_2.5_ removal from the pollution sources. Nano Lett..

[200] Mao X., Si Y., Chen Y., Yang L., Zhao F., Ding B., Yu J. (2012). Silica nanofibrous membranes with robust flexibility and thermal stability for high-efficiency fine particulate filtration. RSC Adv..

[201] Wang Y., Li W., Xia Y., Jiao X., Chen D. (2014). Electrospun flexible self-standing γ-alumina fibrous membranes and their potential as high-efficiency fine particulate filtration media. J. Mater. Chem. A.

[202] Kang Y., Chen J., Feng S., Zhou H., Zhou F., Low Z.X., Zhong Z., Xing W. (2022). Efficient removal of high-temperature particulate matters via a heat resistant and flame retardant thermally-oxidized PAN/PVP/SnO_2_ nanofiber membrane. J. Membr. Sci..

[203] Jia C., Liu Y., Li L., Song J., Wang H., Liu Z., Li Z., Li B., Fang M., Wu H. (2020). A foldable all-ceramic air filter paper with high efficiency and high-temperature resistance. Nano Lett..

[204] Tan J., Zeng Y., Low Z.X., Lin Z., Xu X., Feng S., Zhong Z., Xing W. (2023). Zr-doped flexible TiO_2_ nanofibrous membranes for high-efficiency oily particulate matter removal from high-temperature flue gas. J. Membr. Sci..

[205] Liu K., Liu C., Hsu P.C., Xu J., Kong B., Wu T., Zhang R., Zhou G., Huang W., Sun J. (2018). Core–shell nanofibrous materials with high particulate matter removal efficiencies and thermally triggered flame retardant properties. ACS Cent. Sci..

[206] Zhao T., Teng D., Xu Y., Zhang X., Li Y., Zeng Y. (2022). Multi-functional air filters with excellent flame retardancy and fire-warning capability. J. Colloid Interface Sci..

[207] Lu N., Yan L., Ma Y., Sun H., Zhu Z., Liang W., Li J., Li A. (2022). Electrospun modified SiO_2_ nanofiber membranes as superamphiphobic self-cleaning filters with high heat stability for efficient particle matter capture. ACS Appl. Nano Mater..

[208] Sun W., Liu J., Chu H., Dong B. (2013). Pretreatment and membrane hydrophilic modification to reduce membrane fouling. Membranes.

[209] Petukhov D.I., Johnson D.J. (2024). Membrane modification with carbon nanomaterials for fouling mitigation: A review. Adv. Colloid Interface Sci..

[210] Bhuyan C., Bora P., Rajguru P., Hazarika S. (2025). Thin film nanocomposite membrane ornamented with Z-scheme heterojunction carbon dot/NiFe–LDH for removal of organic contaminants from industrial wastewater. Sep. Purif. Technol..

[211] https://www.researchandmarkets.com/report/hepa-filter.

[212] https://www.gminsights.com/industry-analysis/hvac-filters-market.

[213] Ribeiro B., Vázquez-López A., Vazquez-Pufleau M., Llamosí M., Sempere J., Yuste J., Domenech M., Wang D.Y., Vilatela J.J., Llorca J. (2024). Control of microbial agents by functionalization of commercial air filters with metal oxide particles. Mater. Chem. Phys..

[214] Bächler P., Szabadi J., Meyer J., Dittler A. (2020). Simultaneous measurement of spatially resolved particle emissions in a pilot plant scale baghouse filter applying distributed low-cost particulate matter sensors. J. Aerosol Sci..

[215] Zhang K., Huo Q., Zhou Y.Y., Wang H.H., Li G.P., Wang Y.W., Wang Y.Y. (2019). Textiles/metal–organic frameworks composites as flexible air filters for efficient particulate matter removal. ACS Appl. Mater. Interfaces.

[216] Lee J., Jang J., Kim J., Lim S.H. (2022). A recyclable indoor air filter system based on a photocatalytic metal–organic framework for the removal of harmful volatile organic compounds. Chem. Eng. J..

[217] Reinke F., Meyer J., Dittler A. (2025). Investigation of the potential of in-line particle concentration measurements in gas particle separation processes by using low-cost particulate matter sensors. J. Aerosol Sci..

[218] Lv D., Zhu M., Jiang Z., Jiang S., Zhang Q., Xiong R., Huang C. (2018). Green electrospun nanofibers and their application in air filtration. Macromol. Mater. Eng..

[219] Ángel-Gómez S., Berger S., Niessner J., Álvarez-Láinez M.L. (2024). Air filtration performance of nanofiber membranes with different morphologies produced by green electrospinning. J. Appl. Polym. Sci..

